# Sustainable reduction in sound levels on intensive care units through noise management - an implementation study

**DOI:** 10.1186/s12913-024-12059-9

**Published:** 2025-01-02

**Authors:** Sandra Witek, Claudia Schmoor, Fabian Montigel, Birgit Grotejohann, Sven Ziegler

**Affiliations:** 1 Center of Implementing Nursing Care Innovations Freiburg, Nursing Direction, Medical Center - University of Freiburg, Freiburg, Germany; 2 Clinical Trials Unit, Faculty of Medicine and Medical Center - University of Freiburg, Freiburg, Germany

**Keywords:** Noise, Intensive care units, Implementation science, Intervention, Sound, Noise management

## Abstract

**Background:**

The noise levels in intensive care units usually exceed the recommended limits in (inter)national recommendations. Such noise levels can affect both the recovery of intensive care patients and the performance of staff. The aim of this study was to reduce ward-based noise levels in three intensive care units (anesthesiological, neurological, and neonatological).

**Methods:**

The implementation of a setting-specific intervention bundle consisting of (a) ward-specific guide to noise management, (b) further noise reduction and prevention measures and (c) the use of “noise traffic lights” was evaluated in an implementation study with a pre-post design. Our primary endpoint was changes in sound level (equivalent continuous sound pressure (LAeq)) 12 weeks after the intervention, and the secondary endpoint was sound level (LAeq), peak sound pressure and maximum sound level at different time points, including changes at 24-week follow-up.

**Results:**

After the intervention phase, we observed a significant overall reduction in the sound level of 0.77 decibels (A-weighted) (dB (A)), 95%-CI [0.06, 1.49], *p* = 0.034 with post-intervention measurements of LAeq_1h_ 56.43 dB (A) compared to pre-intervention measurements of 57.21 dB (A). The difference was particularly large (2.21 dB (A) [*p* < 0.0001] in one of the three intensive care units. After adjusting our analysis for the intensity of nursing workload, the sound level reduction was smaller. Comparisons of LAeq_1h_ between measurement times during the daytime periods showed a post-interventional difference of 58.28 dB(A) to 58.84 dB(A) baseline during the day of 0.57 dB(A), 95%-CI [-0.07, 1.21], *p* = 0.08 and at night of 53.36 dB(A) post-interventionally to 54.48 dB(A) baseline a difference of 1.11 dB(A) 95%-CI [0.19, 2.04], *p* = 0.02. In follow-up, baseline sound levels became realigned and we noted a rise in sound level of 0.81 dB (A) [*p* = 0.01].

**Conclusions:**

Our implementation study indicates that a bundle of interventions can reduce noise levels in intensive care units, although the clinical relevance of the measured effect must be questioned. Sufficient resources and a participatory approach using an implementation framework should therefore be employed to manage sustainable noise abatement.

**Trial registration:**

German Clinical Trials Register (DRKS): trial registration number: DRKS00025835; Date of registration: 12.08.2021.

**Supplementary Information:**

The online version contains supplementary material available at 10.1186/s12913-024-12059-9.

## Background

Noise on intensive care units (ICUs) is not just “unwanted noise”.“Noise stress” can trigger acute consequences (e.g., a rise in blood pressure) as well as raise the risk of diseases such as coronary heart disease [1–4]. It can impair the performance and health of intensive care patients and staff alike. For intensive care patients, high noise levels are even associated with delayed recovery [1, 3]. Premature and sick babies are particularly affected by the negative effects of noise on ICUs [5]. However, ICU staff also suffer distraction, memory impairment, and difficulty perceiving warning signals because of high noise levels during complex cognitive tasks [1]. The frequency of false alarms and resulting alarm fatigue are also associated with high noise levels and thus also affect patient safety [6–9]. Relatives are also bothered by noise on ICUs, for example by ambient noise during conversations or negative emotions [1].

The national and international literature contains recommendations on limit values for specific activities and settings. The World Health Organization (WHO) has issued recommendations on environmental noise levels. The latest WHO recommendation specifically referring to the hospital setting dates back to 1999: In patient rooms, an average Equivalent continuous sound pressure (LAeq) of 30 decibels (A-weighted (dB (A)) and maximum sound level (LAmax) of 40 dB (A) should not be exceeded during the day or at night [2]. For more information on sound level measurement terms, see Table 1. It emphasizes that patients (specifically in treatment and monitoring rooms) should be exposed to a maximum average LAeq of 35 dB (A) because of their limited resources for processing stress [1]. For neonatal ICUs, the American Academy of Pediatrics defined limits of 45 dB during the day and 35 dB at night (on average) in 1997 [11]. Most international studies investigating sound levels on ICUs of different disciplines rely on the WHO limits [1, 11–17]. The problem is that these are seldom achievable in acute care settings [13]. Cakir et al. [12] for example, measured an average value of 67.4 dB (A) during the day and 64.8 dB (A) at night. Voigt et al. [15] recorded a mean value of 81 dB (A) in a room with unstable intensive care patients and 61 dB (A) with stable intensive care patients. In any case, it should be possible to achieve a certain reduction from these high levels [13].

**Table 1:** Sound level measurement terminology

Term	Definition
Equivalent continuous sound pressure (Leq)	Leq is the equivalent continuous sound level that represents the total noise exposure over a period of interest or an energy average of the average sound level over the period of interest [10].
peak sound pressure (peak) and Maximum sound level (max)	Peak sound pressure is often confused with maximum sound level. While maximum sound level indicates the highest sound level, the peak sound pressure indicates the highest current value level of the current sound wave. The reason for this is that the maximum level always has time weighting (F, S or I), whereas the peak is the highest value of the sound wave before each evaluation [10].
Fast (F), Slow (S), time weighted	The time weightings F (fast) and S (slow) are defined by the standards (applicable to the devices) and determine the speed at which the device reacts to changes in the sound level. A device set at ‘F’ will respond faster to changes in levels than one set at ‘S’. At a constant sound level, both will yield the same reading. At a dynamic sound level, time weighting can affect the sound level and maximum and minimum values [10].
‘A’ weighted	The ‘A’ rating is a standard rating of hearing frequencies that represents the human ear’s response to sound. The ‘A’ frequency rating is the most widely used to represent the human ear’s response to loudness. Examples are LAeq, LAFmax, where the A indicates the use of A frequency weighting [10].
‘C’ weighted	The ‘C’ ratingyields higher rating for low frequency sounds than the ‘A’ rating does. It is completely flat or linear between 31.5Hz and 8 kHz, the two − 3dB or ‘half power’ point s [10].
Decibel ’A’ weighted dB (A)	The most commonly used frequency weighting to assess the exposure of the human ear to noise (also known as ‘A’ rating or dB (A) [10]).

**Fig. 1:**
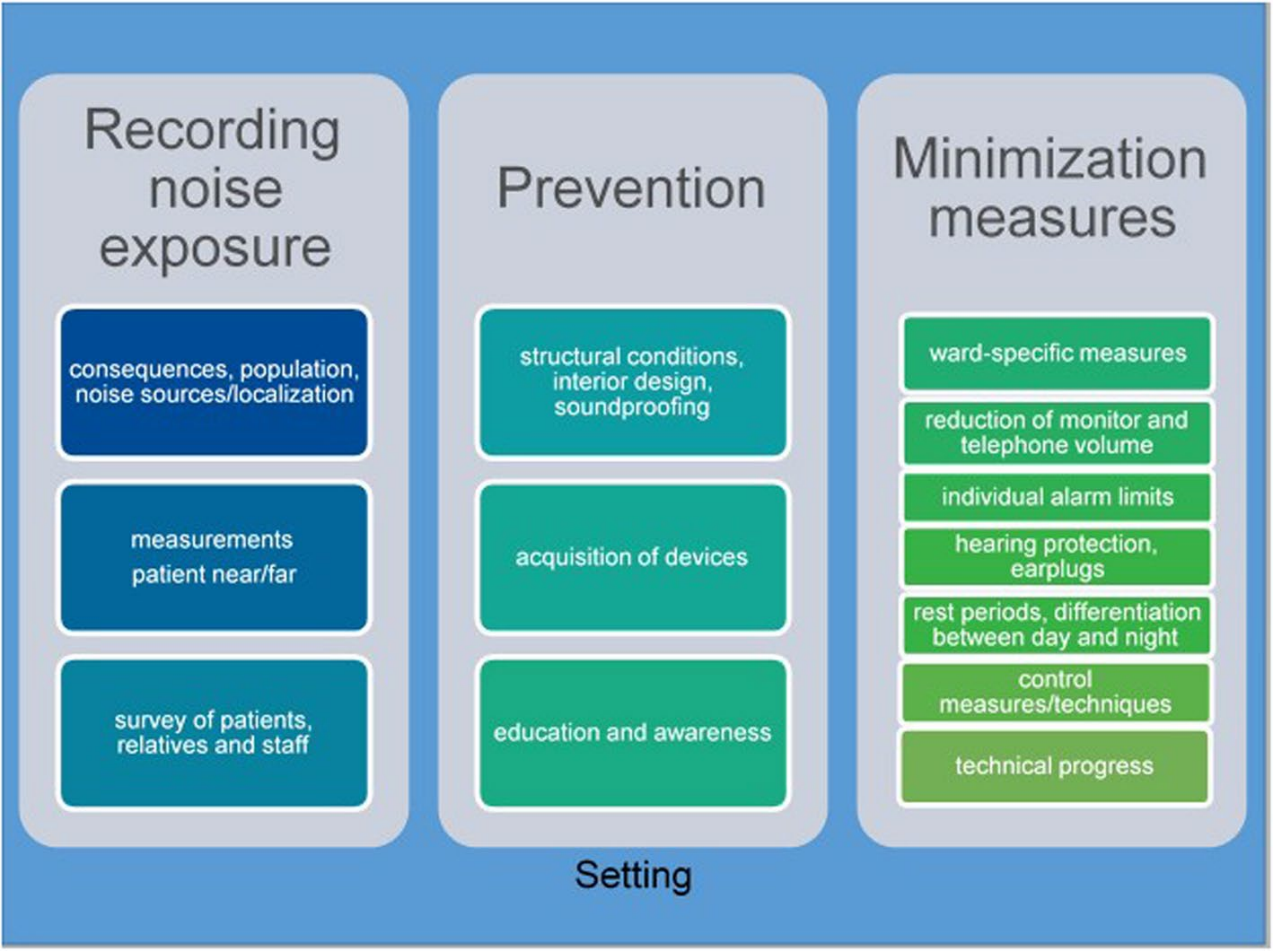
Noise management concept for intensive care units

Despite data from different intervention studies [12, 18–20], there is still a lack of high-quality studies providing evidence-based data on noise-reducing measures on ICUs addressing a long-term effect [22]. Nevertheless, there are indications that intervention bundles are effective [20, 23]. In particular, some researchers have investigated the use of so-called noise traffic lights (NTLs) [23–25]. NTLs are measuring devices that display sound levels or ambient noise visually; they also indicate when it has gotten too loud by emitting an NTL signal [26]. Guisasola-Rabes et al. [25] and Plummer et al. [27] demonstrated significantly lower noise levels in ICUs by using NTLs. Long-term effects have not yet been proven [28]. We unfortunately were unable to find any evidence of effective and long-term noise reduction measures in ICUs in German-speaking countries. However, there are already concepts for reducing noise pollution in a wide variety of work areas (e.g., national standards [29] and the WHO recommendation [1]). The aims of noise management are to promote the assessment and control of noise levels and to optimally protect the health of those affected over the long term [1]. Berglund et al. [1] recommend a step-by-step program to approximate the WHO’s recommended limit values, taking environmental factors into account. Figure 1 illustrates the various measures of the above-mentioned intervention studies. Berglund et al. [1] and the Committee on Industrial Safety [29] emphasize that preventive measures (e.g., educating and informing employees) and noise-reducing measures (e.g., at the noise source itself, or individual measures) must be planned in each setting and for the affected population. Noise management standards are especially recommended [1]. Achievable limit values should be defined and monitored by set measurements. The measures should be taken within a specific time period following an implementation plan and prioritized according to noise focal points and the exposure of those affected (stage-wise program) [1, 29]. The willingness of employees to implement the measures, the setting’s resources, and the people affected must all be taken into account and encouraged [1]. While implementing the measures or the standard, an evaluation should be planned to check its effectiveness and the relationship between costs and benefits [1, 29]. Based on these findings, we conducted a study to help fill the knowledge gap about the long-term effects of noise management bundles and the use of NTLs.

### Aim and objectives

Referring to the chart above, the following research questions arose. (A) To what extent can ward-based noise management in intensive care units sustainably reduce noise levels and noise exposure for intensive care patients and staff? (B) What can NLTs contribute to minimizing noise levels in the long term? Our study’s main objectives were: to minimize noise in ICUs in a sustainable way by implementing noise management in the ICUs concerned, and to sensitize employees to the problem of high noise levels in ICUs and its consequences for intensive care patients and themselves. To this end, we have documented the effects of noise-reducing measures that were monitored and implemented in the participating ICUs.

## Methods

**Fig. 2:**
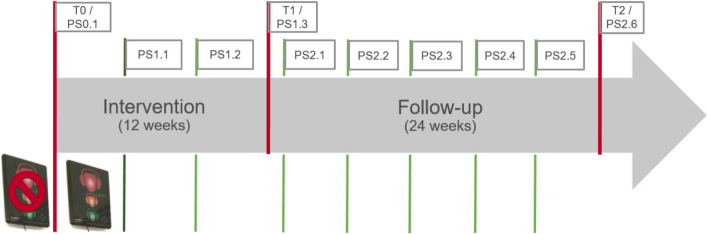
Course of study: illustration. T0 – T2 = Measurement points (sound levels, online surveys, interviews). PS0.1, PS1.1, PS1.2, PS1.3, PS2.1, PS2.2, PS2.3, PS2.4, PS2.5, PS2.6 = Measurement points sound level

This implementation study was a single-center, prospective investigation with a pre-post design and subsequent follow-up. It ran from October 2021 to August 2022 (Fig. 2). We describe this study by applying the “Standards for Reporting Implementation Studies: the StaRI checklist for completion” [30]. Setting.

The three ICUs of different specialties and designs at the University Medical Center Freiburg in Germany investigated in this study were the Anesthesiology ICU (A-ICU), Neonatal ICU (NEO-ICU) and Neurological ICU (NLO-ICU). We selected these ICUs to ensure different characteristics in terms of medical and nursing specialties and the prior identification of noise factors. In the A-ICU with 14 beds, patients of all ages and medical status (e.g. ARDS, perioperative, transplants) are treated (in single to four-bed rooms). The A-ICU has an open design with a central unit around which patient, administrative and social rooms are arranged. The NEO-ICU has 16 beds (single to four-bed rooms) for premature and sick mature babies. The nurses’ station is also centrally positioned here, but a corridor runs parallel to it in which administrative and social rooms are located at a certain distance from the patient rooms. In contrast to the A-ICU and NEO-ICU, the patient rooms in the NLO-ICU (16 beds; single to four-bed rooms) are not arranged around the nurses’ station, but are lined up along a corridor, with the patient rooms on its left-hand side and the staff areas on the right-hand side. The NLO-ICU primarily treats adults with severe, acute neurological conditions (e.g. malignant medial infarction, encephalitis). Because of various years of construction (A-ICU: 2012, NEO-ICU: 2007, NLO-ICU: 1995), the three ICUs’ structures- in addition to their patient populations - differ significantly. In all three ICUs, multi-professional teams (housekeeping and cleaning specialists, medical and pharmaceutical technicians, nursing assistants, nurses, occupational therapists, physicians, physiotherapists, service assistants, speech therapists etc.) contribute to patient care. Nurses usually care for two to three patients during their shift.

### Intervention

One of our research assistants accompanied and supported the ICU teams in implementing noise management. In each ICU, a nurse playing an extended role (nursing manager, advanced practice nurse and Master’s student (nursing science)) was responsible for implementation, while nursing management teams and small (interprofessional) working groups (e.g., with physiotherapists, physicians, nurses) provided support. Online meetings were held monthly during the first half of the study and bi-monthly in the second half to ensure regular communication between those responsible for the ICUs.

The intervention itself consisted of a bundle of different interventions: (1) Development of a ward-specific guide to noise management [1, 29]; (2) further preventive and noise-reducing measures; (3) the use of NTLs.

The ICU managers conducted an initial assessment before initiating the study to identify particularly noisy locations and potential noise sources (noise hotspots). These assessments included a team discussion and inspection of each ICU. Relying on this information and criteria such as visibility for staff, low disturbance for intensive care patients, and the power supply, three NTLs were installed in each ICU. NTLs were installed at the nurses’ station in all three ICUs. Additional NTLs were also installed in four-bed rooms, in the admission area and at the medication counter. The NEO-ICU had the special feature where an NTL microphone was attached onto an incubator. Our assessment findings (e.g., potential noise sources) were incorporated into developing setting-specific guides to noise management and in the planning of preventive and other noise-reducing measures, but were not part of data collection. Preventive and noise-minimizing measures concerned the people involved (staff, intensive care patients, and relatives), the technical equipment and the environment [19]. These guides also relied on those in the literature and current guidelines and included immediate and medium-term measures, or those requiring little or more organizational effort. The setting and patient population were taken into account. The following is a synoptic summary of the measures developed, although not all of them could be implemented during the intervention phase due to the great effort involved:

- Adapting work processes, e.g., restructuring ward rounds.
- Individual alarm management.
- Reacting promptly to alarms, avoiding false alarms.
- Changing infusions and perfusers on time.
- Getting advice from colleagues.
- Introducing rest periods.
- Quieter setting of the ward telephone.
- Conducting telephone calls and conversations outside patients’ rooms whenever possible or quiet conversations in the rooms.
- Reducing the volume of monitors or perfusors, for example, by employees.
- Testing or offering earplugs for (awake) patients or parents.
- Disposing of glass waste outside the patient’s room during night duty.
- Closing doors or leaving them ajar.
- Sending lab samples encased in styrofoam on ICU as a soundproofing measure.
- Avoiding additional noise sources (e.g., radio).
- Placing cushioning on or repair doors, drawers, etc., or purchasing new equipment if necessary.
- Putting rubber studs on chair legs.

Measures in the ward-specific guide to noise management were prioritized [1, 29].

ICU staff were made aware of the noise issue through education and information material from our research team and those responsible in the ICUs. One-Minute-Wonders (posters with specific training content to be completed in one minute) were developed on the consequences for staff of noise and developments in noise levels. Stickers and postcards were also used to inform external employees, visitors, and relatives about the topic. The nursing teams participated in short training sessions on the topic of noise levels and their effects, and on the perspective of intensive care patients and parents of neonatal intensive care patients. In addition, all ICUs underwent weekly evaluations of their average noise levels during the day and night from the intervention phase onwards in order to make the current noise level developments transparent to staff.

The SoundEar® III model from SoundEar A/S was used as an NTL. The advantages of this model are that the data is transmitted to an (internal) cloud (via WLAN), sound levels can be evaluated and the NTL signals’ limits can be set individually - according to the sound level measurements [26]. As visual perception is multifactorial and must be guided by people in a targeted manner, the research team aimed to set the NTL signals so that employees would be sensitized while preventing permanent illumination, which would demotivate employees (e.g., when the cut-off values triggering NTL signals are too proximate) [31, 32]. We programmed and evaluated the NTL signals by relying on sound level measurements in the first third of the intervention phase and during the study course. We have established the following target ranges based on recommendations from the literature to set appropriate alarm thresholds [32] and from an occupational safety expert: NTLs should emit a yellow signal for approximately 7% of the time over a 24-hour period, and a red signal for approximately 2% of the time over a 24-hour period (remainder: green signal). For this purpose, we analyzed the percentage of time when the sound level threshold of x dB (A) (LAeq_1Min_) was exceeded during the day period (06:00–20:59) and night period (21:00–05:59) every measurement point starting from baseline. We set a maximum distance between the yellow and red signal limits of 5 dB. The green signal was switched off in line with a pilot project’s findings (namely that the green signal’s constant illumination probably annoys those present). Table 2 summarizes the NTL settings in the ICUs after our first analysis as an example. Although our aim was to set all the NTLs on an ICU at the same level, this was not always possible because of strong differences in sound levels.

**Table 2:** Setting examples of the traffic light signals

Intensive care unit (ICU)	Signal	During the day (dB (A))	At night (dB (A))	Red signal - delay (seconds (s))	Red signal - duration (s)
A-ICU^a^ at the nurses’ station	yellow	63	58	-	-
	red	67	62	3	5
NEO-ICU^b^ in a four-bed room	yellow	61	58	-	-
	red	64	62	3	5
NLO-ICU^c^ in a four-bed room	yellow	65	63	-	-
	red	70	67	3	5

^a^Anesthesiology ICU

^b^Neonatal ICU

^c^Neurological ICU

The intervention phase consisted of two stages (Fig. 2). In the first third of the intervention phase (PS1.1) (November-December 2021), the sole intervention was to activate and set the NTL signals. The purpose of this intervention was to visualize the noise levels for the employees and separately record any NTL effect (cf. [23, 24]). The NTLs’ signals remained on and active from this point onwards. From the fifth week of the intervention (second third) until the end of the intervention phase (PS1.3) (December 2021 - February 2022), the intervention bundle was implemented by those responsible in the ICUs, accompanied by our research team. However, as there were delays while introducing the guides to noise management, they could only be introduced in the last third of the intervention phase while providing more training and information for staff.

### Data collection

We derived primary and secondary endpoints: primary endpoint was the sound levels in each ICU, measured as the mean sound level LAeq_1Min_, aggregated per hour at the end of the intervention phase (T1 / PS1.3) compared to baseline (T0 / PS0.1). Secondary endpoints were the noise levels in each ICU recorded (over two weeks) in defined time periods during the entire study with the measurements of mean noise level LAeq_1Min_, maximum LAFmax and maximum peak values LCpeakmax includes follow-up at 24 weeks post-intervention. The noise exposure of ICU staff collected via an online survey, and the noise exposure of ICU patients or parents in the neonatal ICU collected via semi-structured interviews at three measurement points each were also linked to the study and have been published elsewhere [33, 34].

To record ambient noise, sound level measurements were taken continuously throughout the study with the individual NTL activated. Measurement locations were those places where NTLs were permanently installed. The baseline survey (T0, PS0.1) was carried out with the NTL switched on and visual feedback switched off. Average sound levels LAeq_1__Min_, maximum values LAFmax and maximum peak values LCpeakmax were recorded according to other studies, the WHO recommendations and the national Noise and Vibration Occupational Safety and Health Ordinance [1, 4, 12, 17, 19, 20, 25, 27, 28, 35].

We took sound level measurements for two weeks each during the study at baseline (phase 0: PS0.1) during the intervention phase (phase 1: PS1.1, PS1.2, PS1.3) and in the follow-up (phase 2: PS2.1, PS2.2, PS2.3, PS2.4, PS2.5, PS2.6). Measurements were taken continuously at two-week intervals from the start of the intervention phase. Apart from the maximum peak values LCpeakmax, the measurements were A-weighted. LCpeakmax were weighted with C. Average sound levels were recorded with LAeq_1Min_. To detect upward deviations, maximum LAFmax per minute was recorded with the time weighting “Fast”. This value must be distinguished from the peak value, as LCpeak records any maximum sound level. The NTL display this as LCpeakmax (per minute).

To record the intensity of the nursing workload in the ICUs, ICU occupancy statistics and documentation of the severity of the patients’ illnesses per ICU were recorded via the intensive care and performance recording system (INPULS^®^) [36]. The INPULS^®^ characterizes the number of patients in 24 h, ventilation minutes, and nursing minutes, which were included in our study’s data analysis.

### Statistical methods

We undertook no formal statistical sample size planning. Planning of the number of ICUs and length of recording periods was based on feasibility considerations. The number of ICUs capable of participating was limited to three. The length of the recording periods was set to 14 days for the following reasons: (a) we considered it sufficient to limit the influence of potential interfering factors on individual days, (b) 14 days were considered as feasible in terms of total study duration, and (c) 14 days are in accordance with earlier studies in this field in which investigation periods were often chosen between 8 and 20 days [25, 27, 37]. The noise level parameters LAeq_1Min_, LAFmax and LCpeakmax were aggregated per hour in all study phases (PS0.1, PS1.1, PS1.2, PS1.3, PS2.1, PS2.2, PS2.3, PS2.4, PS2.5, PS2.6). For LAeq_1Min_, this aggregation was done by calculating the energetic means per hour

$$\:{\text{LAeq}}_{1\text{h}}\:=\:10\:{log}_{10}\:\left[\frac1{60}\:\sum\:_{i=1}^{60}10^{0.1\:{\text{LAeq}}_{1\mathrm{Min}}}\right]$$

For LAFmax and LCpeakmax, aggregation was done by selecting the maximum value per hour.

Our primary endpoint to assess noise reduction was the A-weighted equivalent level LAeq_1h_ as we considered this the most important noise parameter. For our primary assessment of the intervention effect, the LAeq_1h_ measured at the end of the intervention phase (PS1.3) was compared to the LAeq_1h_ measured at baseline (PS0.1). Analysis was performed via a linear regression model for repeated measurements (using PROC MIXED in SAS® 9.4), including the covariates phase (PS1.3 versus (vs.) PS0.1), ICU (A-ICU, NEO-ICU, NLO-ICU), hour, and interactive effects between phase and ICU and between phase and hour. Cluster adjustment was performed within this model by analyzing measurements at the same NTL location per day as repeated measurements modeling a compound symmetric covariance structure assumed to be different between ICUs. A-two-sided *p* value of testing the hypothesis that the intervention effect (PS1.3 vs. PS0.1) is zero was calculated from this model, and the test was performed at the two-sided significance level 0.05. As an estimate of the effect size, the difference in adjusted means (PS1.3 vs. PS0.1) was calculated with two-sided 95%-confidence interval (CI). For both phases, adjusted means per hour with 95%-CI were displayed graphically.

Other secondary endpoints or secondary comparisons between study phases were analyzed applying the same methods. We conducted no alpha adjustment for multiplicity in these analyses. Only our primary analysis result can be interpreted in a confirmatory manner. For all other analyses, *p*-values resulting from statistical tests were interpreted in a descriptive sense.

The secondary endpoints LAFmax and LCpeakmax were compared between PS1.3 and PS0.1. To analyze the short-term effect of the sole activation of the traffic light signal system, we compared noise level parameters at the end of the intervention phase’s first third (PS1.1) to those measured at baseline (PS0.1); to analyze the complete intervention’s long-term effects, we compared noise level parameters at the end of the follow-up phase (PS2.6) to those measured at baseline (PS0.1). In addition to the overall comparisons (all ICUs combined) of noise level parameters between study phases, our results were documented for each of the three ICUs. Mean values of all phases were graphically displayed per hour for all ICUs combined and separately by ICU. Baseline and post intervention phases were also compared separately according to the time of day (day: 06:00 h-20:59 h vs. night: 21:00 h-05:59 h) by including time of day and interaction between time of day and phase as covariates in the regression model.

The intensity of nursing workload on the different ICUs was measured per day by the number of patients, number of care minutes, and number of ventilation minutes. As intensity of nursing workload can also affect noise intensity, were conducted the following analyses we considering potential effects of nursing-workload intensity. The intensity of nursing workload recorded as occupancy parameters were compared between the different phases and ICUs. Analyses of the intervention effects on noise level parameters were adjusted for possible differences in the nursing-workload intensity between study phases and ICUs by including these parameters as covariates in the regression models described above.

## Results

A total of *N* = 1,814,400 sound level measurements were to be taken across all measurement periods and NTL and included in our analysis. During the study, problems with the NTLs’ hardware and software and influencing factors on the ICUs (e.g., power failure, power plug pulled out) triggered incorrect measurements, which we excluded. One of the NEO ICU’s NTLs, which measured the sound levels in an incubator, had to be completely excluded from analysis afterwards, as there were too many faulty measurements and we could not ensure that the microphone had been permanently in an incubator in the designated patient room. Of the remaining eight NTLs, 17,716 measurements (1.1%) had to be excluded.

**Fig. 3:**
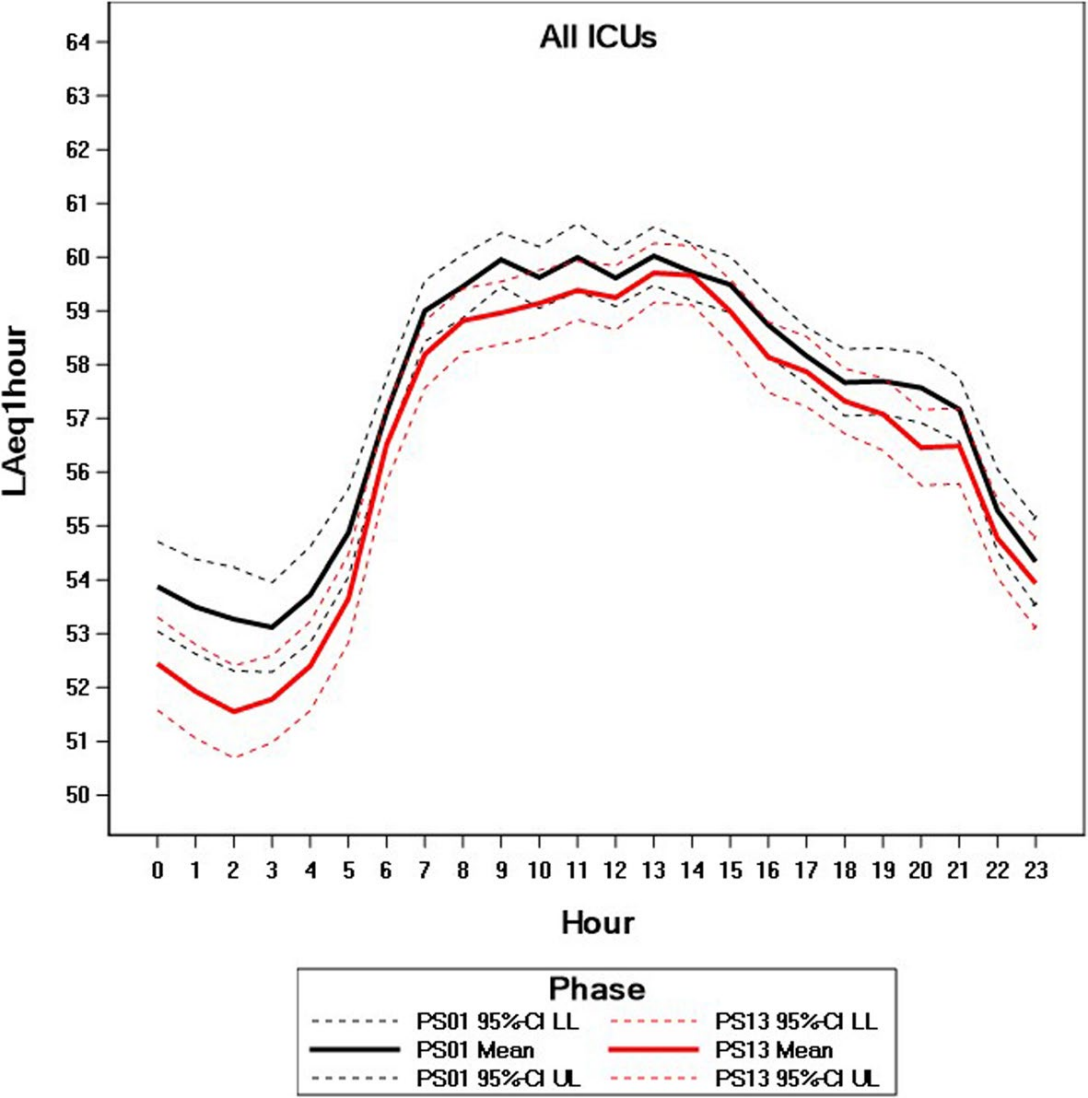
Adjusted means of LAeq_1h_ at baseline and post intervention intervals over 24 hours. ICU: Intensive Care Unit, CI: Confidence interval, PS01: baseline; PS13: post-intervention

Post-interventionally (PS1.3), the overall (all ICUs) mean value (LAeq_1h_) amounted to 56.43 dB (A) [95%-CI: 55.92; 56.94] compared to baseline (PS0.1) of 57.21 dB (A) [95%-CI: 56.71; 57.70]. We recorded a significant reduction of 0.77 dB (A) [95%-CI: 0.06; 1.49; *p* = 0.034] (Table 3). Figure 3 shows the mean values (LAeq_1h_) with 95%-CI over 24 h.

**Table 3:** Adjusted means of LAeq_1h_ at baseline (PS0.1) and post intervention (PS1.3) and differences between adjusted means (PS0.1 vs. PS1.3)

Intensive care unit (ICU)	Time of day	Baseline (PS0.1) (mean dB (A) [95%-CI])	Post-intervention (PS1.3) (mean dB (A) [95%-CI])	Difference PS0.1 vs. PS1.3 (mean dB (A) [95%-CI])	*P*-value
all	all day	57.21 [56.71; 57.70]	56.43 [55.92; 56,94]	0.77 [0.06; 1.49]	0.034
all	day (06:00 h − 20:59 h)	58.84 [58.41; 59.28]	58.28 [57.81; 58.75]	0.57 [−0.07; 1.21]	0.083
all	night (21:00 h − 05:59 h)	54.48 [53.83; 55.12]	53.36 [52.71; 54.02]	1.11 [0.19; 2.04]	0.018
all	difference day vs. night	4.37 [4.01; 4.73]	4.92 [4.51; 5.32]		
A-ICU^a^	all day	57.80 [57.11; 58.49]	57.48 [56.86; 58.09]	0.32 [−0.60; 1.25]	0.49
A-ICU^a^	day (06:00 h − 20:59 h)	59.85 [59.17; 60.53]	59.71 [59.10; 60.33]	0.14 [−0.78; 1.05]	0.77
A-ICU^a^	night (21:00 h − 05:59 h)	54.39 [53.58; 55.20]	53.75 [52.93; 54.58]	0.64 [−0.52; 1.80]	0.28
NEO-ICU^b^	all day	56.51 [55.95; 57.06]	54.30 [53.36; 55.24]	2.21 [1.12; 3.29]	< 0.0001
NEO-ICU^b^	day (06:00 h − 20:59 h)	57.61 [57.05; 58.18]	55.53 [54.62; 56.44]	2.08 [1.01; 3.16]	0.0002
NEO-ICU^a^	night (21:00 h − 05:59 h)	54.66 [54.00; 55.32]	52.25 [51.11; 53.39]	2.41 [1.09; 3.72]	0.0004
NLO-ICU^c^	all day	57,31 [56,11; 58,52]	57,52 [56,48; 58,57]	−0.21 [−1.81; 1.38]	0.79
NLO-ICU^c^	day (06:00 h − 20:59 h)	59.07 [58.10; 60.04]	59.59 [58.71; 60.46]	−0.52 [−1.82; 0.79]	0.44
NLO-ICU^c^	night (21:00 h − 05:59 h)	54.38 [52.74; 56.02]	54.08 [52.71; 55.46]	0.30 [−1.85; 2.44]	0.79

^a^Anesthesiology ICU

^b^Neonatal ICU

^c^Neurological ICU

The daytime mean sound levels LAeq_1h_ fluctuate very strongly. Our comparisons of the mean values (LAeq_1h_) between phases within the times of day yielded a difference post-intervention compared to baseline of 0.57 dB (A) [95%-CI: −0.07; 1.21; *p* = 0.083] during the day and of 1.11 dB (A) [95%-CI: 0.19; 2.04; *p* = 0.018] during the night (Table 3).

**Fig. 4:**
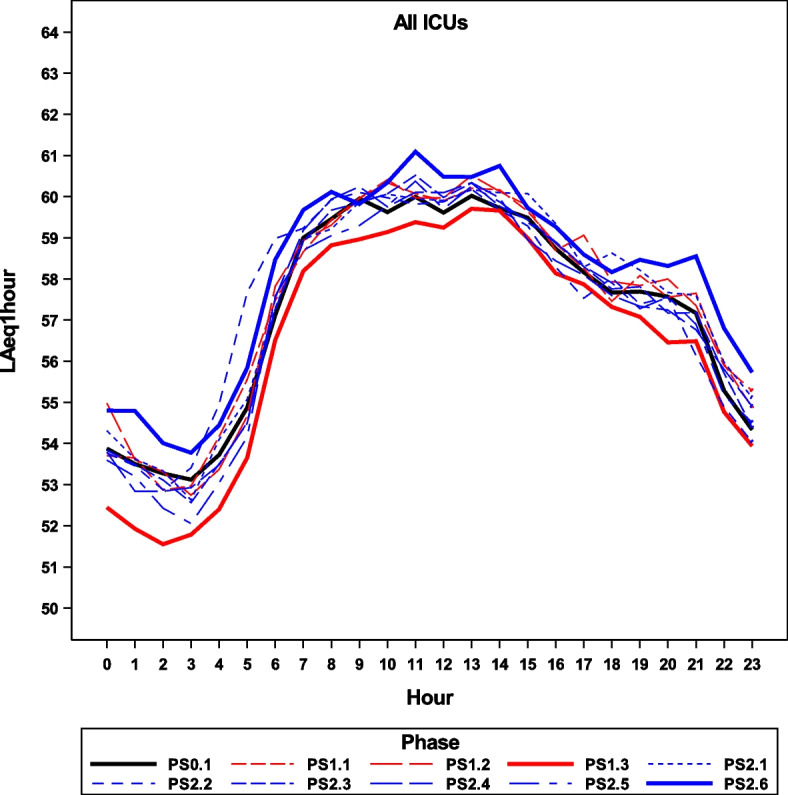
Adjusted mean values (LAeq_1h_) over 24 h of all phases. ICU: Intensive Care Unit; PS01: baseline; PS13: post-intervention; PS2.6: follow-up; PS1.1, PS1.2, PS2.1, PS2.2, PS2.3, PS2.4; PS2.5: between measuring points

Our comparisons of LAeq_1h_ between baseline (PS0.1) and post-intervention (PS1.3) separately on the ICUs is found in Table 3. Note that the baseline to post-intervention reduction is essentially attributable to a reduction in noise levels on the NEO-ICU during the day and at night. There were no differences between the phases - overall or separately within the times of day on the A-ICU and NLO-ICU. Figure 4 shows the mean values (LAeq_1h_) with 95%-CI over 24 h of all phases and all ICUs.

At the end of the first third of the intervention (PS1.1), we observed no relevant difference to baseline that would indicate an influence on the sound levels by sole activation of the NTL signal (Table 4).

**Table 4:** Adjusted means of LAeq_1h_ at baseline (PS0.1) and after first third of intervention phase (PS1.1) and differences between adjusted means (PS0.1 vs.PS1.1)

Intensive care unit (ICU)	Time of day	Baseline (PS0.1) (mean dB (A) [95%-CI])	First third of intervention phase (PS1.1) (mean dB (A) [95%-CI])	Difference PS0.1 vs. PS1.1 (mean dB (A) [95%-CI])	*P*-value
all	all day	57.21 [56.71; 57.70]	57.39 [56.93; 57.86]	−0.19 [−0.87; 0.49]	0.59
all	day (06:00 h − 20:59 h)	58.84 [58.41; 59.28]	58.96 [58.55; 59.37]	−0.11 [−0.71; 0.48]	0.71
all	night (21:00 h − 05:59 h)	54.48 [53.83; 55.12]	54.78 [54.18; 55.38]	−0.31 [−1.19; 0.58]	0.50
all	difference day vs. night	4.37 [4.01; 4.73]	4.18 [3.87; 4.49]		
A-ICU^a^	all day	57.80 [57.11; 58.49]	58.06 [57.45; 58.67]	−0.26 [−1.18; 0.66]	0.58
A-ICU^a^	day (06:00 h − 20:59 h)	59.85 [59.17; 60.53]	60.14 [59.56; 60.72]	−0.29 [−1.18; 0.60]	0.52
A-ICU^a^	night (21:00 h − 05:59 h)	54.39 [53.58; 55.20]	54.61 [53.88; 55.33]	−0.21 [−1.30; 0.87]	0.70
NEO-ICU^b^	all day	56.51 [55.95; 57.06]	56.00 [55.34; 56.66]	0.51 [−0.35; 1.37]	0.25
NEO-ICU^b^	day (06:00 h − 20:59 h)	57.61 [57.05; 58.18]	56.78 [56.15; 57.41]	0.84 [−0.01; 1.68]	0.053
NEO-ICU^b^	night (21:00 h − 05:59 h)	54.66 [54.00; 55.32]	54.69 [53.87; 55.52]	−0.04 [−1.09; 1.02]	0.95
NLO-ICU^c^	all day	57.31 [56,11; 58,52]	58.12 [57.05; 59.18]	−0.81 [−2.41; 0.80]	0.32
NLO-ICU^c^	day (06:00 h − 20:59 h)	59.07 [58.10; 60.04]	59.96 [59.09; 60.84]	−0.89 [−2.20; 0.42]	0.18
NLO-ICU^c^	night (21:00 h − 05:59 h)	54.38 [52.74; 56.02]	55.05 [53.62; 56.47]	−0.67 [−2.84; 1.51]	0.55

^a^Anesthesiology ICU

^b^Neonatal ICU

^c^Neurological ICU

After the follow-up (PS2.6), LAeq_1h_ was 58.02 dB (A) [95%-CI: 57.68; 58.36], which shows a difference from the baseline of −0.81 [−1.42; −0.21], *p* = 0.008, and thus an increase in the sound level. As these differences vary at the ward level, the increase in sound level is primarily attributable to the NLO-ICU. Table 5 summarizes the mean values LAeq_1h_ post-follow-up compared to the baseline overall, at the ward level and at the times of day.

**Table 5:** Adjusted means of LAeq_1h_ at baseline (PS0.1) and post follow-up (PS2.6) and differences between adjusted means (PS0.1 vs. PS2.6)

Intensive care unit (ICU)	Time of day	Baseline (PS0.1) (mean dB (A) [95%-CI])	Post-follow-up (PS2.6) (mean dB (A) [95%-CI])	Difference PS0.1 vs. PS2.6 (mean dB (A) [95%-CI])	*P*-value
all	all day	57.21 [56.71; 57.70]	58.02 [57.68; 58.36]	−0.81 [−1.42; −0.21]	0.008
all	day (06:00 h − 20:59 h)	58.84 [58.41; 59.28]	59.48 [59.14; 59.82]	−0.64 [−1.19; −0.08]	0.025
all	night (21:00 h − 05:59 h)	54.48 [53.83; 55.12]	55.59 [55.18; 55.99]	−1.11 [−1.87; −0.35]	0.005
all	difference day vs. night	4.37 [4.01; 4.73]	3.89 [3.60; 4.19]		
A-ICU^a^	all day	57.80 [57.11; 58.49]	57.39 [56.73; 58.04]	0.42 [−0.53; 1.37]	0.39
A-ICU^a^	day (06:00 h − 20:59 h)	59.85 [59.17; 60.53]	59.47 [58.85; 60.09]	0.38 [−0.754; 1.30]	0.42
A-ICU^a^	night (21:00 h − 05:59 h)	54.39 [53.58; 55.20]	53.91 [53.10; 54.72]	0.48 [−0.67; 1.63]	0.41
NEO-ICU^b^	all day	56.51 [55.95; 57.06]	55.94 [55.21; 56.67]	0.56 [−0.35; 1.48]	0.23
NEO-ICU^b^	day (06:00 h − 20:59 h)	57.61 [57.05; 58.18]	56.69 [55.90; 57.48]	0.93 [−0.05; 1.90]	0.062
NEO-ICU^b^	night (21:00 h − 05:59 h)	54.66 [54.00; 55.32]	54.70 [53.99; 55.42]	−0.05 [−1.02; 0.93]	0.93
NLO-ICU^c^	all day	57.31 [56,11; 58,52]	60.73 [60.47; 60.99]	−3.42 [−4.65; 2.19]	< 0.0001
NLO-ICU^c^	day (06:00 h − 20:59 h)	59.07 [58.10; 60.04]	62.29 [62.08; 62.52]	−3.21 [−4.21; 2.22]	< 0.0001
NLO-ICU^c^	night (21:00 h − 05:59 h)	54.38 [52.74; 56.02]	58.14 [57.59; 58.69]	−3.76 [−5.49; −2.03]	< 0.0001

^a^Anesthesiology ICU

^b^Neonatal ICU

^c^Neurological ICU

Qualitatively, the LAFmax and LCpeakmax results resemble those for LAeq_1h_. They also initially reveal a sound level reduction that approached the baseline again in the follow-up and even exceeded it (Table 6 + 7). This appears in more detail in the supplemental appendix (Supplemental file 1).

**Table 6:** Adjusted means of LAFmax and LCpeakmax at baseline (PS0.1) and post-intervention (PS1.3) and differences between adjusted means (PS0.1 vs. PS1.3)

All intensive care units	Baseline (PS0.1) (mean dB (A/C) [95%-CI])	Post-intervention (PS1.3) (mean dB (A/C) [95%-CI])	Difference PS0.1 vs. PS1.3 (mean dB (A/C) [95%-CI])	*P*-value
LAFmax	82.77 [82.19; 83.36]	82.13 [81.55; 82.71]	0.64 [−0.18; 1.47]	0.12
LCpeakmax	101.51 [100.93; 102.08]	100.83 [100.28; 101.38]	0.68	0.09

**Table 7:** Adjusted means of LAFmax and LCpeakmax at baseline (PS0.1) and post-follow-up (PS2.6) and differences between adjusted means (PS0.1 vs. PS2.6)

All intensive care units	Baseline (PS0.1) (mean dB (A/C) [95%-CI])	Post-follow-up (PS2.6) (mean dB (A/C) [95%-CI])	Difference PS0.1 vs. PS2.6 (mean dB (A/C) [95%-CI])	*P*-value
LAFmax	82.77 [82.19; 83.36]	83.56 [83.05; 84.08]	−0.79 [−1.57; −0.01]	0.047
LCpeakmax	101.51 [100.93; 102.08]	101.95 [101.44; 102.46]	−0.44 [−1.21; 0.33]	0.26

Table 8 shows the number of patients in 24 h, nursing minutes per day, and ventilation minutes per day at each measurement time. These varied between both the wards and between measurement times. We observed that the noise intensity is affected by the number of patients in 24 h and nursing minutes in particular. For the LAeq_1h_ post-interventionally (PS1.3) to baseline (PS0.1) the mean LAeq_1h_ increased per additional patient by 0.25 dB (A) [95%-CI: 0.04; 0.45], *p* = 0.02, and per additional 1000 nursing minutes by 0.34 dB (A) [95%-CI: 0.04; 0.64], *p* = 0.028. In analyzing our comparison for the primary outcome (comparison PS0.1. vs. PS1.3), adjusted for the number of patients, we noted a smaller reduction in LAeq_1h_ of 0.58 dB (A) [95%-CI: −0.16; 1.31], *p* = 0.12, compared to the unadjusted analysis, as the number of patients in PS1.3 was lower than in PS0.1. Thus, after adjusting the comparisons for the number of patients in 24 h, ventilation minutes and nursing minutes per day, we observed smaller differences in the mean values LAeq_1h_ post-interventionally, after the first third of the intervention and post-follow-up to baseline overall, and at ward level (Table 9). Additional file 1 contains detailed adjusted analyses of the mean LAFmax and LCpeakmax. They also show overall smaller differences than without adjusting for the intensity of nursing workload.

**Table 8:** Comparison of occupancy parameters at baseline (PS0.1), 1st third of the intervention (PS1.1), post-intervention (PS1.3) and post-follow-up (PS2.6)

Measurement times	A-ICU^a^	NEO-ICU^b^	NLO-ICU^c^	All ICU
Number of patients in 24 h mean [95%-CI]				
Baseline (PS0.1)	13.4 [12.6; 14.2]	16.1 [15.3; 16.9]	13.9 [13.1; 14.7]	14.5 [14.0; 15.0]
First third of intervention (PS1.1)	14.8 [14.0; 15.5]	15.9 [15.2; 16.7]	15.1 [14.3; 15.8]	15.3 [14.8; 15.7]
Post-intervention (PS1.3)	13.8 [13,0; 14,6]	12.1 [11.3; 12.9]	15.2 [14.4; 16.0]	13.7 [13.2; 14.2]
Post-follow-up (PS2.6)	15.1 [14,3; 16,0]	14,3 [13,5; 15,1]	15.1 [14.3; 16.0]	14.9 [14.4; 15.3]
Ventilation minutes mean [95%-CI]				
Baseline (PS0.1)	11,467 [10411; 12524]	11,648 [10591; 12705]	4486 [3429; 5543]	9201 [8594; 9807]
First third of intervention (PS1.1)	11,141 [10084; 12197]	9898 [8841; 10955]	6789 [5733; 7846]	9276 [8666; 9886]
Post-intervention (PS1.3)	7797 [6747; 8847]	5199 [4149; 6249]	7380 [6330; 8430]	6792 [6186; 7398]
Post-follow-up (PS2.6)	7820 [6554; 9086]	9399 [8133; 10665]	9653 [8387; 10919]	8957 [8226; 9688]
Nursing minutes mean [95-%-CI]				
Baseline (PS0.1)	10,250 [9774; 10726]	11,282 [10806; 11758]	7896 [7393; 8346]	9800 [9525; 10076]
First third of intervention (PS1.1)	11,157 [10681; 11634]	11,801 [11325; 12277]	9058 [8581; 9534]	10,672 [10397; 10947]
Post-intervention (PS1.3)	8406 [7929; 8883]	7124 [6647; 7601]	9562 [9085; 10039]	8364 [8089; 8640]
Post-follow-up (PS2.6)	9472 [8939; 10005]	10,315 [9782; 10848]	10,221 [9668; 10754]	10,003 [9695; 10310]

^a^Anesthesiology ICU

^b^Neonatal ICU

^c^Neurological ICU

**Table 9:** Adjusted differences of LAeq_1h_ at ward level additionally adjusted for possible differences in nursing-workload intensity

Intensive care unit	Time of day	Difference PS0.1 vs. first third of intervention PS1.1 (mean dB (A) [95%-CI], *p*-value)	Difference PS0.1 vs. PS1.3 (mean dB(A) [95%-CI], *p*-value)	Difference PS0.1 vs. PS2.6 (mean dB(A) [95%-CI], *p*-value)
Adjustment according to patient numbers in 24 h				
all	all day	0.04 [−0.65; 0.72], *p* = 0.92	0.58 [−0.16; 1.31], *p* = 0.12	−0.73 [−1.33; −0.14], *p* = 0.02
A-ICU^a^	all day	0.14 [−0.82; 1.09], *p* = 0.78	0.41 [−0.51; 1.33], *p* = 0.38	0.81 [−0.17; 1.80], *p* = 0.11
NEO-ICU^b^	all day	0.45 [−0.38; 1.27], *p* = 0.29	1.22 [−0.09; 2.53], *p* = 0.077	0.13 [−0.81; 1.07], *p* = 0.78
NLO-ICU^c^	all day	−0.47 [−2.08; 1.14], *p* = 0.56	0.10 [−1.49; 1.70], *p* = 0.90	−3.14 [−4.36; −1.97], *p* < 0.0001
Adjustment according to ventilation minutes				
all	all day	−0.18 [−0.86; 0.50], *p* = 0.60	0.57 [−0.23; 1.36], *p* = 0.16	−0.82 [−1.42; −0.21], *p* = 0.01
A-ICU^a^	all day	−0.28 [−1.21; 0.64], *p* = 0.55	0.01 [−1.07; 1.0], *p* = 0.98	0.37 [−0.66; 1.40], *p* = 0.48
NEO-ICU^b^	all day	0.40 [−0.48; 1.28], *p* = 0.37	1.66 [−0.26; 3.06], *p* = 0.020	0.53 [−0.41; 1.47], *p* = 0.26
NLO-ICU^c^	all day	−0.66 [−2.29; 0.97], *p* = 0.43	0.03 [−1.62; 1.68], *p* = 0.97	−3.36 [−4.70; −2.01], *p* < 0.0001
Adjustment according to care minutes				
all	all day	0.06 [−0.66; 0.79], *p* = 0.86	0.29 [−0.55; 1.12], *p* = 0.504	−0.79 [−1.40; −0.19], *p* = 0.01
A-ICU^a^	all day	−0.00 [−0.96; 0.96], *p* = 0.10	−0.30 [−1.44; 0.84], *p* = 0.60	0.33 [−0.64; 1.31], *p* = 0.50
NEO-ICU^b^	all day	0.66 [−0.20; 1.51], *p* = 0.13	0.79 [−0.81; 2.39], *p* = 0.33	0.46 [−0.48; 1.40], *p* = 0.34
NLO-ICU^c^	all day	−0.47 [−2.11; 1.18], *p* = 0.58	0.36 [−1.32; 2.04], *p* = 0.67	−3.17 [−4.56; −1.77], *p* < 0.0001

^a^Anesthesiology ICU

^b^Neonatal ICU

^c^Neurological ICU

## Discussion

We observed an overall reduction in sound levels after the intervention phase. However, the differences varied primarily at ward level. At the end of the study, sound levels converged with baseline. Overall, we detected a rise in the sound level post-follow-up, particularly on the NLO-ICU. Intensity of nursing workload recorded as occupancy parameters differed at the measurement times; patient numbers in 24 h and nursing minutes revealed particular influence on sound levels. After adjusting for the occupancy parameters, the post-intervention sound level reduction was smaller.

The WHO recommendations were nowhere nearly achieved post-interventionally with the mean value (LAeq_1h_) 56.43 dB (A) [95-%-CI: 55.92; 56.94] and maxima of LAFmax 82.13 dB (A) [95-%-CI: 81.55; 82.71], thus confirming reports that they are practically unachievable on ICUs [1, 13]. Although our NEO-ICU results indicate a sound level reduction during the day (difference: 2.08 dB (A), 95%-CI [1.01; 3.16], *p* = 0.0002) and at night (difference: 2.41 dB (A), 95-%-CI [1.09; 3.72], *p* = 0.0004), the sound levels are still above the American Academy of Pediatrics recommendation [10]. We noted particularly at the end of the study (post-follow-up) that the effects recorded post-interventionally approached the baseline again and even exceeded it. Overall, our results underline the findings of Voigt et al. [15] (in contrast to the intervention studies [18–20]) as ours reveal the influence of occupancy and the level of patient care on sound levels in ICUs according to the severity of the patients’ illnesses. These influencing factors can therefore distort potential sound level reductions after interventions have taken place, as Vreman et al. [22] confirm. Wang et al. [24] adjusted the sound levels according to intensive therapy activities in particular. However, Litton et al. [28] were again unable to demonstrate a correlation between LAmax and occupancy rates.

The scope and focus of the interventions examined in studies varies considerably. Christofel et al. [21] for example, focused solely on the ventilators. Crawford et al. [19] developed behavior-based intervention bundles. The intervention bundle by Kol et al. [20] addressed even more aspects concerning the interprofessional team, premises and equipment on an ICU. The scope of our study’s intervention bundle falls somewhere between Crawford et al. [19] and Kol et al. [20]. Despite a fundamentally equally interprofessional approach (e.g., in working groups), nurses on the ICUs were primarily involved. The intervention phases of Crawford et al. [19] and Kol et al. [20] were shorter than our study’s three-month intervention phase. However, due to the delays described above, our intervention phase’s shorter duration must be assumed.

Kol et al. [20] is the only one of the three aforementioned intervention studies [18–20] demonstrating a significant sound level reduction, thus proving the effectiveness of their intervention bundle. They demonstrated a sustained effect [20], in contrast to the present study. Nevertheless, we assume that bundles should include interventions for employees, equipment, and the environment (environmental change) [19]. Vreman et al. [22] emphasize that an environmental change can facilitate behavioral change among employees by adapting the structural conditions. In the present study, however, we could only address environmental change incompletely because of project specifications and the ICU staff’s initiative. Enabling sustainable behavioral changes in employees in noise management terms seems to be primarily achievable with complex interventions or strategies having multiple components [22]. Despite this evidence, Wang et al. [24], Guisasola-Rabes et al. [25] and Plummer et al. [27] investigated the sole use of NTLs (different models) in various ICUs (e.g., neonatology) and concluded that activated NTLs can indeed influence human behavior. In the present study, the use of NTLs was considered as part of the intervention bundle, but we were unable to evaluate any isolated NTL effects in the first third of the intervention.

Despite the above-mentioned international recommendations and concepts, sound level measurement procedures differ from one study to another. Ours relied primarily on the WHO recommendations [1] and operational safety guidelines [29]. Vreman et al. [22] expand on those recommendations. In principle, sound levels are measured A-weighted [4, 11, 12, 14–20, 26]. Only a few studies report on peak values [25, 27] and these were also recorded using C-weighting [27]. In the present study, we examined different measured values to be able to depict a basic sound level (LAeq), maximum (LAFmax) and very short and loud sound levels (LCpeak) [cf. [25]]. However, heterogeneous measurements across studies means that our results can only be selectively compared to the aforementioned studies, nor are those investigations themselves comparable.

Certain measurement locations were often close to the patients’ heads in studies [11, 12, 14–20, 26] often or close to the nurses’ station to enable the depiction and if necessary comparison of perspectives of patients and staff. Studies [4, 12, 13] also specify the position of the microphone or measuring device and potentially influencing factors such as distances to walls or devices. We followed the producer’s specifications regarding such details [26], and the NTLs’ visual aspect was usually decisive. Although we aimed to place the microphone in the patients’ immediate vicinity (in the incubator), finding a permanent position in the incubator was not possible, which is why we had to exclude those measurements. NTLs were installed in the four-bed rooms on all three ICUs - farther away from the patients than in the studies described at the beginning of this section. Litton et al. [28] took a fundamentally different approach, as they wanted to make sound level measurements possible in everyday inpatient life using a clinically accessible tool (app for iOS and Android).

The settings themselves - the ICUs with their special features - are very heterogeneous in all the studies mentioned, in line with international conditions. In the present study we also revealed clear differences in structural conditions and patient populations between ICUs. In particular, Song et al. [17], Crawford et al. [19] and Voigt et al. [15] make it clear that the sound level measurements cannot be compared when the number of beds per room differs and when hospital environment and structural conditions all differ. Only one ICU was examined in most of the published studies addressing noise levels [4, 11, 12, 16–19]. Studies involving several ICUs - like ours - are rare [16]. The present study stands out for having investigated ICUs with adults, all age groups, and premature/ill term infants. Note Litton et al. [28] in this context, they examined 39 ICUs in their cross-sectional observational study. Nevertheless, more research involving several different ICUs is needed to increase comparability.

In studies investigating NTLs, the threshold values set for the NTL signals differ significantly according to the setting where they were recorded (Table 10). Guisasola-Rabes et al. [25] and Wang et al. [23] did not differentiate between daytime and nighttime (as we did), and it is unclear whether these devices are even capable of doing so. They also relied on baseline data (deactivated NTLsignal) as a reference value for setting the NTL signals [25].

**Table 10:** Comparison of noise traffic light signals’ threshold values in three studies [23–25] and our settings study

	Wang et al. [24] (Phase 1/2)	NEO^b^-ICU (during the day, at night)	Guisasola et al. [25] (all day)	A^a^-/NLO^c^-ICU (during the day, at night)	Plummer et al. [27] (at night; off during the day)
yellow signal	40/45 dB	58–61 dB (A)	55 dB (A)	58–65 dB (A)	60 dB (A)
red signal	45/50 dB	62–64 dB (A)	60 dB (A)	62–70 dB (A)	70 dB (A)

^a^Anesthesiology ICU

^b^Neonatal ICU

^c^Neurological ICU

The clinical relevance of the magnitude of sound level reductions has only been occasionally discussed [19, 25]. However, it is important, because decibels have a logarithmic structure. Accordingly, a drop in the sound pressure level by 3 dB equals a “halving of the sound intensity” [38]. Small sound level reductions were achieved by Crawford et al. [19] and Guisasola-Rabes et al. [24] and in our investigation, and some were nevertheless statically significant. Crawford et al. [19] question the clinical relevance of a sound level reduction of < 1 dB (A), as it is an imperceptible difference to the human ear. Darbyshire and Duncan Young [39] also recommend a threshold difference of at least 3 dB for clinical relevance for those reasons. Nevertheless, these arguments, Guisasola-Rabes et al. [25] suggest that a minor reduction in sound level may still be clinically relevant in ICUs (e.g., sleep quality). With a post-interventional reduction of 0.77 dB, our primary outcome must be viewed critically in terms of clinical relevance, as must the slight increase over time (in our study and [25]) In any case, we were unable to demonstrate a clinically relevant effect from employing NTLs in our study. More research here is obviously warranted.

### Strengths and limitations

Despite applying our intervention bundle as a means of noise management, we could not ascertain the extent to which its actual implementation was influenced by each approach to the planning, introduction, and implementation of noise management in ICUs by ICU staff, and by their motivation.As the implementation of noise management involves multiple measures, the inclusion of a framework for developing and evaluating complex interventions such as the Medical Research Council (MRC) guidance would have been helpful to plan the measures and better understand their interactions. An iterative approach should be emphasized it would have enabled a structured and continuous adaptation of noise management measures to meet real-world needs [40]. Furthermore, the incorporation of a conceptual framework for the translation of research into practice would have been advantageous. For the structured planning and development of individualized noise management concepts, Intervention Mapping (IM) can support the development of a systematic approach for evidence-based and target-group specific interventions [41]. Moreover, the integrated Promoting Action on Reasearch Implementation in Health Services (i-PARIHS) Framework could also be helpful as a theoretical concept to address the challenges of research translation [42]. At the same time, our study’s general conditions were a limiting factor. A randomized contrrolled trial was not feasble as our study could be conducted in only three ICUs. Due to the participating ICUs’ technical and structural differences, we would have needed to randomize more ICUs between intervention and control in order to differentiate the intervention’s effects from those of an ICU’s structure and setting. Despite thorough preparation, the ten-month trial period was somewhat too brief to carry out the implementation. The process of approving the setting-specific guidelines within the hospital, for example, led to delays. Another factor that could have influenced the implementation is probably the unilateral involvement of staff with various qualifications (e.g., in planning noise-reducing measures, or training) on each ICU (see above). Environmental change (e.g., spatial or medical technology conditions) could not be thoroughly examined because of the nature of our study’s position within a third-party funded project. However, our study’s design and implementation only enabled us to reveal incompletely the process behind changing ICU staff behavior. The NTLs’ successful implementation can be questioned, as they had to be monitored primarily by our research team during the study as there was no independent management of this technology on the ICUs. Nevertheless, the NTLs were implemented in everyday patient care. Their hardware and software (e.g., log file, export, data backup via USB stick) make supporting, maintaining, and evaluating them very time-consuming. Despite regular contact with their manufacturer and sales department, we were unable to easily handle the NTLs (e.g., especially data analysis, Cloud) for the ICUs even after the study.

### Implications

Our study findings reveal that a participatory and sustainable implementation approach is needed to effectively manage noise levels on an ICU. In future investigations of implementing noise management on ICUs, researchers will need to consider the motivation of the staff and other professionals, and the facilities and equipment in the setting, as these are decisive factors to be ascertained in advance when planning and implementing such noise managment. A flexible timetable entailing priorities for implementation is recommended. Our study results prompted us to launch a follow-up study a year later to investigate the adherence of staff in ICUs and potential influencing factors for noise management in greater depth. That data is now being analyzed.

## Conclusions

We failed to demonstrate a noise reduction via individual measures, including the sole use of NTLs. Our research findings nevertheless suggest that intervention bundles targeting ward-based noise management are potential means of reducing noise levels on ICUs. The technologies for visualizing and measuring noise levels in ICUs, however, seem to need improvement. However, both the noise issue and implementation processes are complex. To gain well-founded insights into these factors in the future, more research is needed on the intervention bundle, the implementation processes entailing validated and objective measurements, and on the documentation of the subjective experiences of different groups on ICUs.

## Supplementary Information

Supplementary Material 1.

## Data Availability

The datasets generated and/or analyzed during the current study are not publicly available but are available from the corresponding author on reasonable request.

## Abbreviations

A-ICU: Anesthesiology intensive care unit
CI: Confidence Interval
dB (A)/(C): decibels A- or C-weighted
ICU(s): Intensive care unit(s)
IM: Intervention Mapping
INPULS®: intensive care and performance recording system
i-PARIHS: Reasearch Implementation in Health Services
MRC: Medical Research Council
NEO-ICU: Neonatal intensive care unit
NLO-ICU: Neurological intensive care unit
NTL: Noise traffic light
s: second(s)
WHO: World Health Organization
