# Supplementary material for: Sustainable reduction in sound levels on intensive care units through noise management - an implementation study
Source: BMC Health Serv Res. 2025 Jan 2;25:9. doi: 10.1186/s12913-024-12059-9 (PMC11697813; doi:10.1186/s12913-024-12059-9)
Supplement: Supplementary file 1 — Supplementary Material 1. [file 12913_2024_12059_MOESM1_ESM.pdf]

Witek, S, Schmoor, C, Montigel, F, Grotejohann, B, Ziegler, S.

Sustainable reduction in sound levels on intensive care units through noise management - an implementation study

Supplementary File

2024-12-10

Table of Contents

Table 1 - Mean LAFmax baseline (PS0.1) and 1st third of intervention (PS1.1) compared to the time of day and at ward level [95%-confidence interval (CI), p-value] 2

Table 2 - Mean LAFmax baseline (PS0.1) and post-intervention (PS1.3) compared to the time of day and at ward level [95%-confidence interval (CI), p-value] 4

Table 3 - Mean LAFmax baseline (PS0.1) and post-follow-up (PS2.6) compared to the time of day and at ward level [95%-confidence interval (CI), p-value] 6

Table 4 - Mean LCpeakmax baseline (PS0.1) and 1st intervention third (PS1.1) compared to the time of day and at ward level [95%-confidence interval (CI), p-value 8

Table 5 - Mean LCpeakmax baseline (PS0.1) and post-intervention (PS1.3) compared to the time of day and at ward level [95%-confidence interval (CI), p-value], at ward level 10

Table 6 - Mean LCpeakmax baseline (PS0.1) and post-follow-up (PS2.6) compared to the time of day and at ward level [95%-confidence interval (CI), p-value], at ward level 12

Table 7 - Adjusted differences of LAFmax baseline (PS0.1) vs. 1st third of intervention (PS1.1), baseline (PS0.1) vs. post-interventional (PS1.3) and baseline (PS0.1) vs. follow-up (PS2.6) [95%-confidence interval (CI), p-value,], at ward level 14

Table 8 - Adjusted differences of LCpeakmax baseline (PS0.1) vs. 1st third of intervention (PS1.1), baseline (PS0.1) vs. post-interventional (PS1.3) and baseline (PS0.1) vs. follow-up (PS2.6) [95%-confidence interval (CI), p-value,], at ward level 16

Figure 1 - Mean LAFmax baseline and post-intervention 18

Figure 2 - Mean LAFmax baseline and post-follow-up 19

Figure 3 - Mean LAFmax of all measurement points 20

Figure 4 - Average maximum values LAFmax at ward level (A-ICU) 21

Figure 5 - Average maximum values LAFmax at ward level (NEO-ICU) 22

Figure 6 - Average maximum values LAFmax at ward level (NLO-ICU) 23

Figure 7 - Mean, maximum peak values LCpeakmax baseline and post-intervention 24

Figure 8 - Mean, maximum peak values LCpeakmax baseline and post-follow-up 25

Figure 9 - Average, maximum peak values LCpeakmax with all measurement points 26

Figure 10 - Average, maximum peak values LCpeakmax of all measurement points at ward level (A-ICU) 27

Figure 11 - Average, maximum peak values LCpeakmax of all measurement points at ward level (NEO-ICU) 28

Figure 12 - Average, maximum peak values LCpeakmax of all measurement points at ward level (NLO-ICU) 29

Table 1 - Mean LAFmax baseline (PS0.1) and 1st third of intervention (PS1.1) compared to the time of day and at ward level [95%-confidence interval (CI), p-value]

**Intensive care unit** (ICU)	**Time of day**	**Baseline (PS0.1)** (mean dB (A) [95 %-CI])	**1st Intervention-third (PS1.1)** (mean dB (A) [95 %-CI])	**Difference PS0.1 versus PS1.1** (mean dB (A) [95 %-CI])	**P-value**
all	all day	82.13 [81.55; 82.71]	82.91 [82.35; 83.48]	-0.14 [-0.95; 0.67]	0.73
all	day (06:00 h – 20:59 h)	84.22 [83.68; 84.76]	84.32 [83.82; 84.83]	-0.10 [-0.84; 0.63]	0.79
all	night (21:00 h – 05:59 h)	80.36 [79.61; 81.11]	80.57 [79.83; 81.30]	-0.21 [-1.26; 0.85]	0.70
all	difference day versus night	3.86 [3.41; 4.31]	3.75 [3.32; 4.19]		
A-ICU*	all day	83.93 [82.88; 84.98]	84.24 [83.21; 85.27]	-0.31 [−1.78; 1.16]	0.68
A-ICU*	day (06:00 h – 20:59 h)	85.49 [84.51; 86.43]	85.99 [85.03; 86.95]	-0.50 [-1.87; 0.88]	0.48
A-ICU*	night (21:00h – 05:59 h)	81.32 [80.03; 82.60]	81.31 [80.05; 82.57]	0.01 [−1.79, 1.81]	0.99
NEO-ICU**	all day	80.99 [80.18; 81.81]	80.46 [79.62; 81.30]	0.54 [-0.63; 1.71]	0.38
NEO-ICU**	day (06:00 h – 20:59 h)	81.88 [81.04; 82.72]	81.24 [80.41; 82.06]	0.64 [-0.53; 1.82]	0.28
NEO-ICU**	night (21:00 h – 05:59 h)	79.52 [78.65; 80.39]	79.16 [78.15; 80.18]	0.36 [-0.98; 1.70]	0.60
NLO-ICU***	all day	83.40 [82.24; 84.55]	84.05 [83.00; 85.09]	-0.65 [-2.21; 0.91]	0.41

NLO-ICU***	day (06:00 h – 20:59 h)	85.29 [84.32; 86.25]	85.74 [84.91; 86.57]	−0.45 [−1.72; 0.82]	0.48
NLO-ICU***	night (21:00 h – 05:59 h)	80.25 [78.61; 81.88]	81.23 [79.72; 82.73]	-0.98 [−3.20; 1.24]	0.38

* Anaesthesiology ICU, **Neonatal ICU, ***Neurological ICU

Table 2 - Mean LAFmax baseline (PS0.1) and post-intervention (PS1.3) compared to the time of day and at ward level [95%-confidence interval (CI), p-value]

**Intensive care unit** (ICU)	**Time of day**	**Baseline (PS0.1)** (mean dB (A) [95 %-CI])	**Post-intervention (PS1.3)** (mean dB (A) [95 %-CI])	**Difference PS0.1 versus PS1.3** (mean dB (A) [95 %-CI])	**P-value**
all	all day	82.13 [81.55; 82.71]	82.77 [82.19; 83.36]	0,64 [-0.18; 1.47]	0.12
all	day (06:00 h – 20:59h)	84.22 [83.68; 84.76]	83.73 [83.22; 84.23]	0.49 [-0.24; 1.23]	0.19
all	night (21:00 h – 05:59 h)	80.36 [79.61; 81.11]	79.46 [78.67; 80.26]	0.90 [-0.19; .1.99]	0.11
all	difference day versus night	3.86 [3.41; 4.31]	4.27 [3.75; 4.78]		
A-ICU*	all day	83.93 [82.88; 84.98]	84.03 [83.04; 85.01]	-0.10 [−1.54; 1.34]	0.89
A-ICU*	day (06:00 h – 20:59 h)	85.49 [84.51; 86.43]	85.72 [84.79; 86.64]	-0.22 [-1.58; 1.13]	0.75
A-ICU*	night (21:00 h – 05:59 h)	81.32 [80.03; 82.60]	81.21 [79.97; 82.46]	0.10 [−1.69; 1.90]	0.91

NEO-ICU**	all day	80.99 [80.18; 81.81]	78.78 [77.75; 79.81]	2.21 [0.90; 3.53]	0.001
NEO-ICU**	day (06:00 h – 20:59 h)	81.88 [81.04; 82.72]	79.89 [79.03; 80.75]	1.99 [0.79; 3.19]	0.001
NEO-ICU**	night (21:00 h – 05:59 h)	79.52 [78.65; 80.39]	76.94 [75.49; 78.38]	2.58 [0.90; 4.27]	0.003
NLO-ICU***	all day	83.40 [82.24; 84.55]	83.58 [82.60; 84.56]	-0.18 [-1.70; 1.34]	0.81
NLO-ICU***	day (06:00 h – 20:59 h)	85.29 [84.32; 86.25]	85.58 [84.75; 86.41]	−0.29 [−1.56; 0.97]	0.65
NLO-ICU***	night (21:00 h – 05:59 h)	80.25 [78.61; 81.88]	80.24 [78.82; 81.66]	0.01 [−2.16; 2.17]	0.99

* Anaesthesiology ICU, **Neonatal ICU, ***Neurological ICU

Table 3 - Mean LAFmax baseline (PS0.1) and post-follow-up (PS2.6) compared to the time of day and at ward level [95%-confidence interval (CI), p-value]

**Intensive care unit** (ICU)	**Time of day**	**Baseline (PS0.1)** (mean dB (A) [95 %-CI])	**Post-follow-up (PS2.6)** (mean dB (A) [95 %-CI])	**Difference PS0.1 versus PS2.6** (mean dB (A) [95 %-CI])	**P-value**
all	all day	82.13 [81.55; 82.71]	83.56 [83.05; 84.08]	-0.79 [-1.57; -0.01]	0.047
all	day (06:00 h – 20:59 h)	84.22 [83.68; 84.76]	84.86 [84.37; 85.34]	-0.64 [-1.36; 0.09]	0.086
all	night (21:00 h – 05:59 h)	80.36 [79.61; 81.11]	81.41 [80.76; 82.06]	-1.05 [-2-04; 0.05]	0.039
all	difference day versus night	3.86 [3.41; 4.31]	3.45 [3.00; 3.89]		
A-ICU*	all day	83.93 [82.88; 84.98]	83.77 [82.69; 84.86]	0.16 [−1.54; 1.34]	0.84
A-ICU*	day (06:00 h – 20:59h)	85.49 [84.51; 86.43]	85.55 [84.53; 86.56]	-0.05 [-1.47; 1.36]	0.94
A-ICU*	night (21:00 h – 05:59 h)	81.32 [80.03; 82.60]	80.82 [79.50; 82.13]	0.50 [−1.34; 2.34]	0.59
NEO-ICU**	all day	80.99 [80.18; 81.81]	80.26 [79.23; 81.30]	0.73 [-0.59; 2.05]	0.28
NEO-ICU**	day (06:00 h – 20:59 h)	81.88 [81.04; 82.72]	80.94 [79.94; 81.94]	0.94 [-0-37; 2.24]	0.16
NEO-ICU**	night (21:00 h – 05:59 h)	79.52 [78.65; 80.39]	79.14 [77.92; 80.36]	0.38 [-1.12; 1.88]	0.62
NLO-ICU***	all day	83.40 [82.24; 84.55]	86.65 [86.31; 87.00]	-3.26 [-4.47; -2.05]	<.0001

NLO-ICU***	day (06:00 h – 20:59 h)	85.29 [84.32; 86.25]	88.08 [87.74; 88.42]	−2.80 [−3.82; -1.78]	<.0001
NLO-ICU***	night (21:00 h – 05:59 h)	80.25 [78.61; 81.88]	84.27 [83.50; 85.04]	-4.03 [−5.83; -2.22]	<.0001

* Anaesthesiology ICU, **Neonatal ICU, ***Neurological ICU

Table 4 - Mean LCpeakmax baseline (PS0.1) and 1st intervention third (PS1.1) compared to the time of day and at ward level [95%-confidence interval (CI), p-value

**Intensive care unit** (ICU)	**Time of day**	**Baseline (PS0.1)** (mean dB (C) [95 %-CI])	**Post-intervention (PS1.1)** (mean dB (C) [95 %-CI])	**Difference PS0.1 versus PS1.1** (mean dB (C) [95 %-CI])	**P-value**
all	all day	101.51 [100.93; 102.08]	101.72 [101.17; 102.57]	-0.21 [-1.00; 0.58]	0.60
all	day (06:00 h – 20:59 h)	103.00 [102.46; 103.55]	103.12 [102.61; 103.62]	-0.11 [-0.86; 0.63]	0.76
all	night (21:00 h – 05:59h)	99.02 [98.27; 99.76]	99.39 [98.68; 100.10]	-0.37 [-1.40, 0.66]	0.48
all	difference day versus night	3.98 [3.47; 4.50]	3.73 [3.27; 4.18]		
A-ICU*	all day	103.06 [101.89; 104.24]	103.60 [102.45; 104.75]	-0.54 [−2.18; 1.11]	0.52
A-ICU*	day (06:00 h – 20:59h)	104.57 [103.46; 105.68]	105.27 [104.20; 106.34]	-0.70 [-2.24; 0.84]	0.37
A-ICU*	night (21:00 h – 05:59 h)	100.55 [99.10; 102.01]	100.82 [99.43; 102.21]	-0.26 [−2.27; 1.75]	0.80
NEO-ICU**	all day	99.37 [98.58; 100.15]	98.84 [98.06; 99.62]	0.53 [-0.57; 1.64]	0.34

NEO-ICU**	day (06:00 h – 20:59 h)	100.31 [99.45; 101.18]	99.64 [98.81; 100.46]	0.67 [-0.52; 1.87]	0.27
NEO-ICU**	night (21:00 h – 05:59 h)	97.80 [97.00; 98.60]	97.50 [96.60; 98.40]	0.30 [-0.91; 1.50]	0.63
NLO-ICU***	all day	102.10 [101.11; 103.08]	102.72 [101.84; 103.60]	-0.63 [-1.94; 0.69]	0.35
NLO-ICU***	day (06:00 h – 20:59 h)	104.13 [103.31; 104.94]	104.44 [103.75; 105.14]	−0.32 [−1.39; 0.76]	0.56
NLO-ICU***	night (21:00 h – 05:59 h)	98.71 [97.21; 100.21]	99.85 [98.51; 101.18]	-1.14 [−3.15; 0.87]	0.26

* Anaesthesiology ICU, **Neonatal ICU, ***Neurological ICU

Table 5 - Mean LCpeakmax baseline (PS0.1) and post-intervention (PS1.3) compared to the time of day and at ward level [95%-confidence interval (CI), p-value], at ward level

**Intensive care unit** (ICU)	**Time of day**	**Baseline (PS0.1)** (mean dB (C) [95 %-CI])	**Post-intervention (PS1.3)** (mean dB (C) [95 %-CI])	**Difference PS0.1 versus PS1.3** (mean dB (C) [95 %-CI])	**P-value**
all	all day	101.51 [100.93; 102.08]	100.83 [100.28; 101.38]	0.68 [-0.12; 1.47]	0.094
all	day (06:00 h – 20:59 h)	103.00 [102.46; 103.55]	102.49 [102.00; 102.98]	0.52 [-0.21; 1.25]	0.17
all	night (21:00 h – 05:59 h)	99.02 [98.27; 99.76]	98.07 [97.30; 98.84]	0.95 [-0.12; 2.02]	0.082
all	difference day versus night	3.98 [3.47; 4.50]	4.42 [3.87; 4.97]		
A-ICU*	all day	103.06 [101.89; 104.24]	103.07 [101.97; 104.18]	-0.01 [−1.62; 1.60]	0.99
A-ICU*	day (06:00 h – 20:59 h)	104.57 [103.46; 105.68]	104.70 [103.66; 105.75]	-0.14 [-1.66; 1.39]	0.86
A-ICU*	night (21:00 h – 05:59 h)	100.55 [99.10; 102.01]	100.36 [98.95; 101.76]	0.20 [−1.82; 2.22]	0.85
NEO-ICU**	all day	99.37 [98.58; 100.15]	97.03 [96.14; 97.92]	2.34 [1.15; 3.53]	0.0001
NEO-ICU**	day (06:00 h – 20:59 h)	100.31 [99.45; 101.18]	98.36 [97.59; 99.14]	1.95 [0.78; 3.11]	0.001

NEO-ICU**	night (21:00 h – 05:59 h)	97.80 [97.00; 98.60]	94.80 [93.53; 96.07]	3.00 [1.50; 4.50]	0.0001
NLO-ICU***	all day	102.10 [101.11; 103.08]	102.39 [101.54; 103.23]	-0.29 [-1.59; 1.00]	0.67
NLO-ICU***	day (06:00 h – 20:59 h)	104.13 [103.31; 104.94]	104.39 [103.71; 105.07]	−0.26 [−1.33; 0.80]	0.62
NLO-ICU***	night (21:00 h – 05:59 h)	98.71 [97.21; 100.21]	99.05 [97.72; 100.38]	-0.34 [−2.35; 1.67]	0.74

* Anaesthesiology ICU, **Neonatal ICU, ***Neurological ICU

Table 6 - Mean LCpeakmax baseline (PS0.1) and post-follow-up (PS2.6) compared to the time of day and at ward level [95%-confidence interval (CI), p-value], at ward level

**Intensive care unit** (ICU)	**Time of day**	**Baseline (PS0.1)** (mean dB (C) [95%-CI])	**Follow-up (PS2.6)** (mean dB (C) [95%-CI])	**Difference PS0.1 versus PS2.6** (mean dB (C) [95%-CI])	**P-value**
all	all day	101.51 [100.93; 102.08]	101.95 [101.44; 102.46]	-0.44 [-1.21; 0.33]	0.26
all	day (06:00 h – 20:59 h)	103.00 [102.46; 103.55]	103.39 [102.90; 103.88]	-0.38 [-1.11; 0.35]	0.30
all	night (21:00 h – 05:59 h)	99.02 [98.27; 99.76]	99.56 [98.90; 100.22]	-0.54 [-1.54; 0.45]	0.28
all	difference day versus night	3.98 [3.47; 4.50]	3.82 [3.36; 4.29]		
A-ICU*	all day	103.06 [101.89; 104.24]	103.16 [101.99; 104.33]	-0.10 [−1.76; 1.56]	0.99
A-ICU*	day (06:00 h – 20:59h)	104.57 [103.46; 105.68]	105.17 [104.05; 106.28]	-0.60 [-2.17; 0.97]	0.45
A-ICU*	night (21:00 h – 05:59 h)	100.55 [99.10; 102.01]	99.82 [98.42; 101.22]	0.74 [−1.28; 2.75]	0.47
NEO-ICU**	all day	99.37 [98.58; 100.15]	98.30 [97.39; 99.21]	1.07 [-0.14; 2.27]	0.082
NEO-ICU**	day (06:00 h – 20:59 h)	100.31 [99.45; 101.18]	99.09 [98.20; 99.98]	1.22 [-0.02; 2.47]	0.053

NEO-ICU**	night (21:00 h – 05:59 h)	97.80 [97.00; 98.60]	96.99 [95.87; 98.11]	0.80 [-0.57; 2.18]	0.25
NLO-ICU***	all day	102.10 [101.11; 103.08]	104.39 [103.99; 104.79]	-2.30 [-3.36; -1.23]	<.0001
NLO-ICU***	day (06:00 h – 20:59 h)	104.13 [103.31; 104.94]	105.90 [105.56; 106.24]	−1.77 [−2.66; -0.89]	0.0001
NLO-ICU***	night (21:00 h – 05:59 h)	98.71 [97.21; 100.21]	101.87 [101.04; 102.71]	-3.17 [−4.88; -1.45]	0.0003

* Anaesthesiology ICU, **Neonatal ICU, ***Neurological ICU

Table 7 - Adjusted differences of LAFmax baseline (PS0.1) vs. 1st third of intervention (PS1.1) , baseline (PS0.1) vs. post-interventional (PS1.3) and baseline (PS0.1) vs. follow-up (PS2.6) [95%-confidence interval (CI), p-value,], at ward level

**Intensive care unit** (ICU)	**Time of day**	**Difference PS0.1 vs. PS1.1** (mean dB (A) [95 %-CI], p-value)	**Difference PS0.1 vs. PS1.3** (mean dB (A) [95 %-CI], p-value)	**Difference PS0.1 vs. PS2.6** (mean dB (A) [9 5%-CI], p-value)
**adjustment according to number of patients in 24 h**				
all	all day	0.00 [-0.83; 0.83], p = 0.99	0.49 [-0.37; 1.36], p = 0.26	-0.73 [-1.50; 0.05], p = 0.067
A-ICU*	all day	-0.05 [−1.55; 1.45], p = 0.95	-0.03 [−1.46; 1.40], p = 0.97	0.47 [−1.07; 2.01], p = 0.55
NEO-ICU**	all day	1.44 [-0.67; 1.66], p = 0.40	1.44 [-0.26; 3.14], p = 0.096	0.39 [-0.94; 1.72], p = 0.57
NLO-ICU***	all day	-0.44 [−2.01; 1.14], p = 0.59	0.07 [−1.47; 1.60], p = 0.93	-3.03 [−4.24; -1.83], p <.0001
**adjustment according to ventilation minutes**				
all	all day	-0.14 [-0.95; 0.68], p = 0.74	0.50 [-0.44; 1.43], p = 0.30	-0.78 [-1.56; 0.00], p = 0.050
A-ICU*	all day	-0.33 [−1.80; 1.14], p = 0.66	-0.33 [−1.93; 1.27], p = 0.69	0.33 [−1.27; 1.93], p = 0.70
NEO-ICU**	all day	0.43 [-0.76; 1.63], p = 0.48	1.81 [0.04; 3.59], p = 0.045	0.84 [-0.53; 2.20], p = 0.23

NLO-ICU***	all day	-0.51<br>[−2.13; 1.10],<br>p = 0.53	-0.00<br>[−1.61; 1.61],<br>p = 0.99	-3.50<br>[−4.92; -2.08],<br>p <.0001
**adjustment according to care minutes**				
all	all day	0.01<br>[-0.88; 0.91],<br>p = 0.97	0.34<br>[-0.72; 1.41],<br>p = 0.52	-0.79<br>[-1.58; -0.01],<br>p = 0.05
A-ICU*	all day	-0.15<br>[−1.66; 1.36],<br>p = 0.85	-0.49<br>[−2.20; .1.23],<br>P = 0.58	0.17<br>[−1.37; 1.71],<br>p = 0.83
NEO-ICU**	all day	0.63<br>[-0.56; 1.82],<br>p = 0.30	1.34<br>[-0.91; 3.60],<br>p = 0.24	0.75<br>[-0.60; 2.10],<br>p = 0.28
NLO-ICU***	all day	-0.44<br>[−2.08; 1.20],<br>p = 0.60	0.17<br>[−1.53; 1.87],<br>p = 0.84	-3.30<br>[−4.80; -1.81],<br>p <.0001

* Anesthesiology ICU, **Neonatal ICU, ***Neurological ICU

Table 8 - Adjusted differences of LCpeakmax baseline (PS0.1) vs. 1st third of intervention (PS1.1) , baseline (PS0.1) vs. post-interventional (PS1.3) and baseline (PS0.1) vs. follow-up (PS2.6) [95%-confidence interval (CI), p-value,], at ward level

**Intensive care unit** (ICU)	**Time of day**	**Difference PS0.1 vs. PS1.1** (mean dB (A) [95 %-CI], p-value)	**Difference PS0.1 vs. PS1.3** (mean dB(A) [95 %-CI], p-value)	**Difference PS0.1 vs. PS2.6** (mean dB(A) [95 %-CI], p-value)
**adjustment according to number of patients in 24 h**				
all	all day	-0.06 [-0.87; 0.74], p = 0.89	0.50 [-0.33; 1.33], p = 0.24	-0.35 [-1.11; 0.41], p = 0.37
A-ICU*	all day	-0.28 [−1.95; 1.40], p = 0.75	0.07 [−1.54; 1.69], p = 0.93	0.34 [−1.35; 2.04], P = 0.69
NEO-ICU**	all day	0.49 [-0.60; 1.59], p = 0.38	1.42 [-0.11; 2.95], p = 0.069	0.59 [-0.60; 1.78], p = 0.33
NLO-ICU***	all day	-0.41 [−1.73; 0.91], p = 0.54	0.00 [−1.30; 1.30], p = 0.99	-1.98 [−3.02; -0.95], p = 0.0002
**adjustment according to ventilation minutes**				
all	all day	-0.21 [-1.00; 0.59], p = 0.61	0.65 [-0.23; 1.53], p = 0.15	-0.42 [-1.19; 0.35], p = 0.28
A-ICU*	all day	0.54 [−2.19; 1.11], p = 0.52	-0.05 [−1.78; 1.68], p = 0.95	0.22 [−1.50; 1.93], p = 0.80
NEO-ICU**	all day	0.51 [-0.62; 1.63], p = 0.38	2.27 [0.71; 3.83], p = 0.005	1.26 [0.03; 2.49], p = 0.044

NLO-ICU***	all day	-0.59<br>[−1.96; 0.78],<br>p = 0.40	-0.26<br>[−1.65; 1.13],<br>p = 0.71	-2.75<br>[−4.00; -1.49],<br>p <.0001
**adjustment according to care minutes**				
all	all day	-0.10<br>[-09.97; 0.77],<br>p = 0.82	0.48<br>[-0.54; 1.50],<br>p = 0.36	-0.45<br>[-1.23; 0.32],<br>p = 0.25
A-ICU*	all day	-0.42<br>[−2.10; 1.26],<br>p = 0.62	-0.27<br>[−2.10; 1.56],<br>p = 0.77	-0.05<br>[−1.74; 1.63],<br>p = 0.95
NEO-ICU**	all day	0.60<br>[-0.53; 1.72],<br>p = 0.30	1.76<br>[-0.38; 3.90],<br>p = 0.11	1.12<br>[-0.11; 2.35],<br>p = 0.073
NLO-ICU***	all day	-0.47<br>[−1.88; 0.94],<br>p = 0.51	-0.06<br>[−1.54; 1.43],<br>p = 0.94	-2.43<br>[−3.78; -1.08],<br>p = 0.0005

* Anaesthesiology ICU, **Neonatal ICU, ***Neurological ICU

Figure 1 - Mean LAFmax baseline and post-intervention

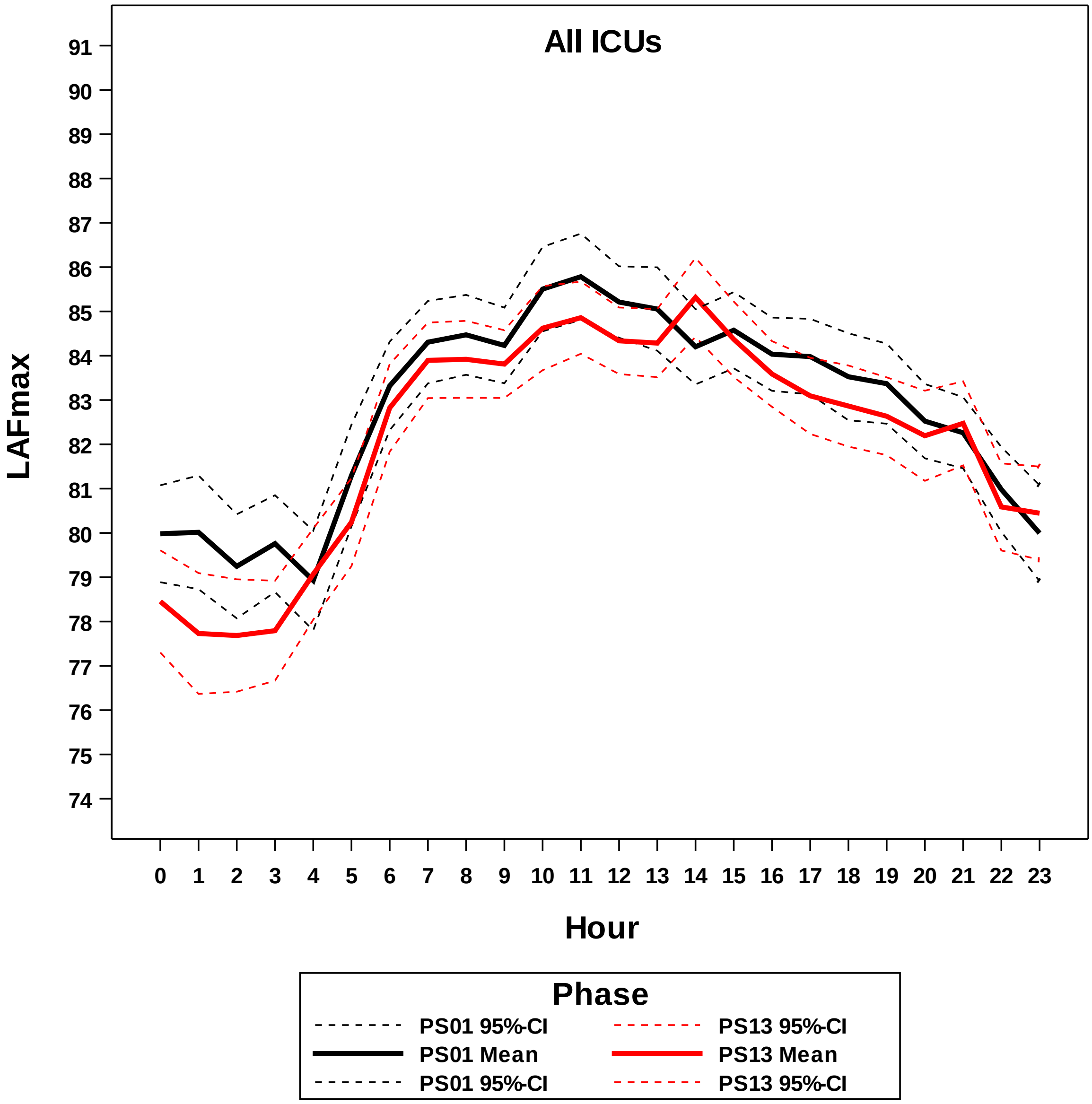

* ICU: Intensive Care Unit, CI: Confidence interval, PS01: baseline; PS13: post-intervention

Figure 2 - Mean LAFmax baseline and post-follow-up

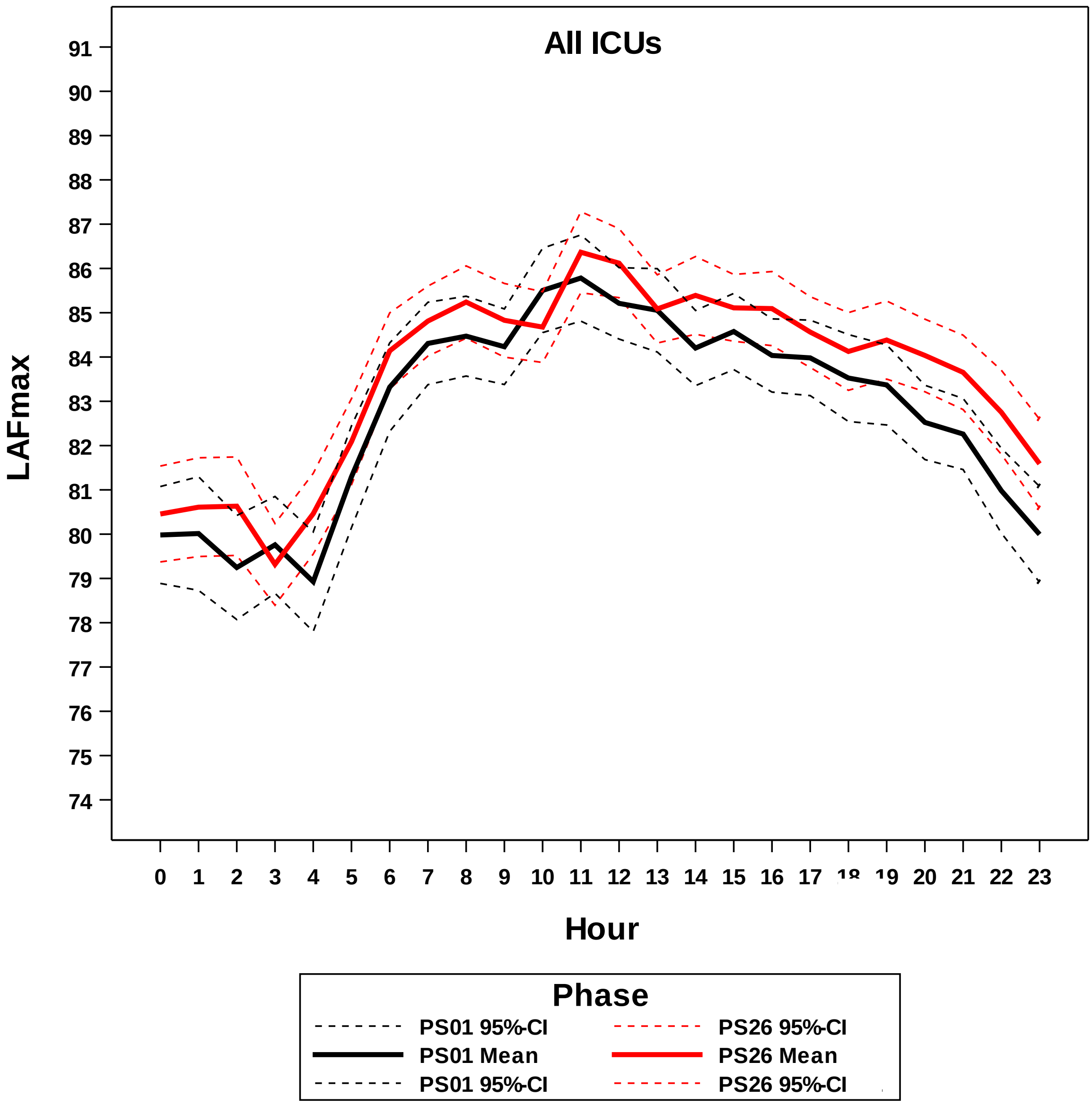

* ICU: Intensive Care Unit; PS01: baseline; PS13; PS2.6: follow-up; CI: Confidence Interval

Figure 3 - Mean LAFmax of all measurement points

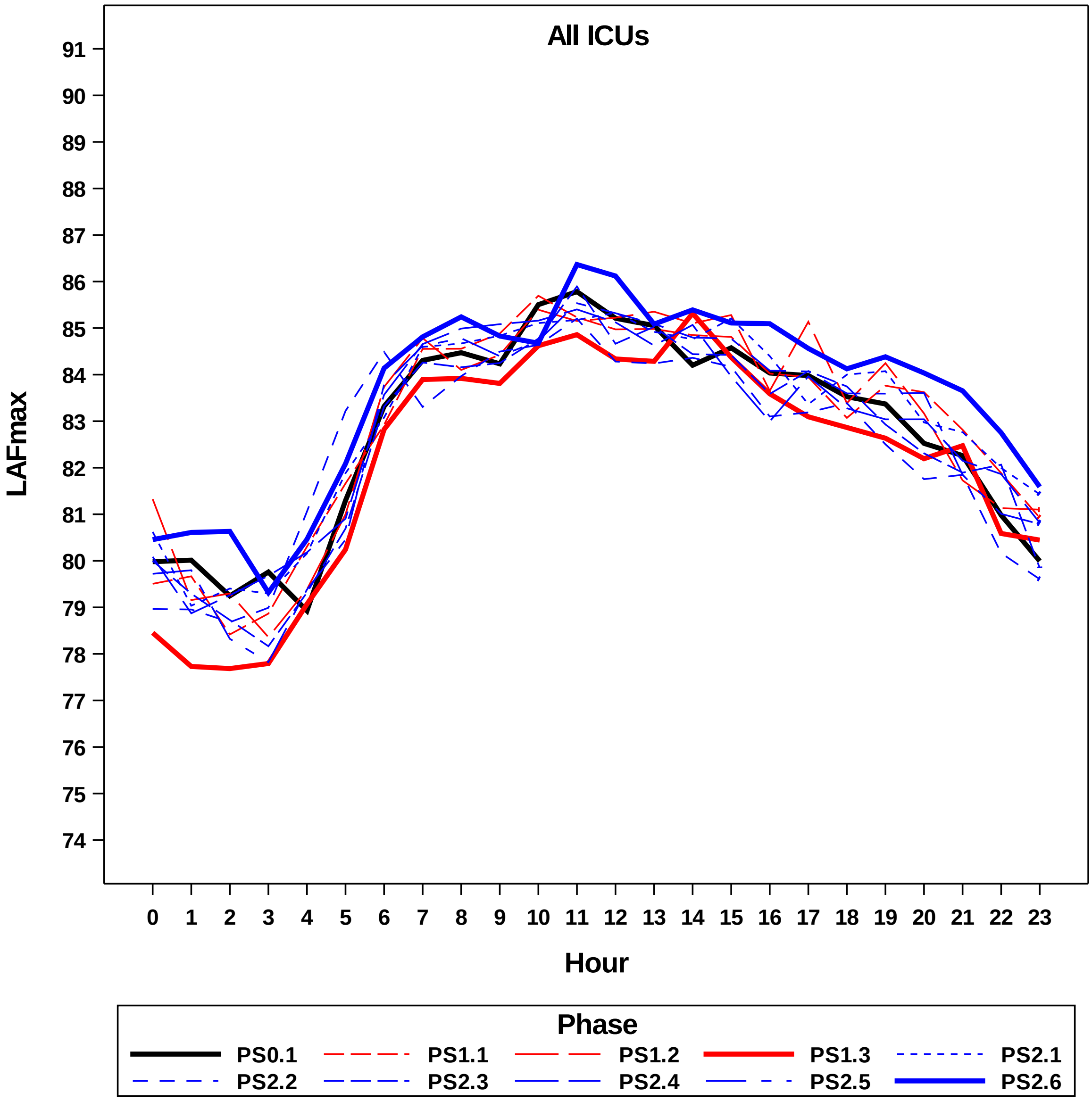

* ICU: Intensive Care Unit; PS01: baseline; PS13: post-intervention; PS2.6: follow-up; PS1.1, PS1.2, PS2.1, PS2.2, PS2.3, PS2.4; PS2.5: between measuring points

Figure 4 - Average maximum values LAFmax at ward level (A-ICU)*

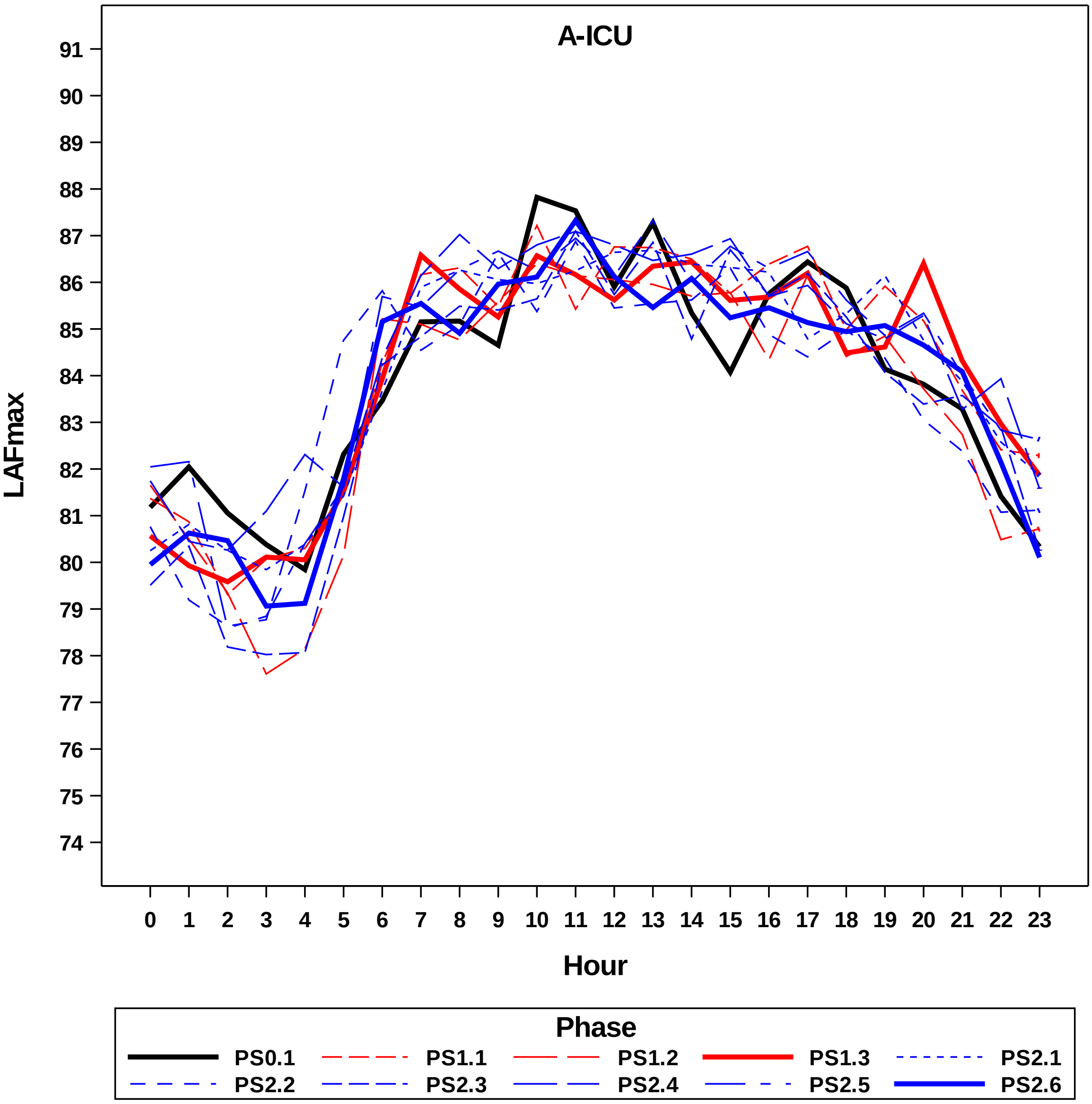

* Anaesthesiology intensive care unit: PS01: baseline; PS13: post-intervention; PS2.6: follow-up;

PS1.1, PS1.2, PS2.1, PS2.2, PS2.3, PS2.4; PS2.5: between measuring points

Figure 5 - Average maximum values LAFmax at ward level (NEO-ICU)*

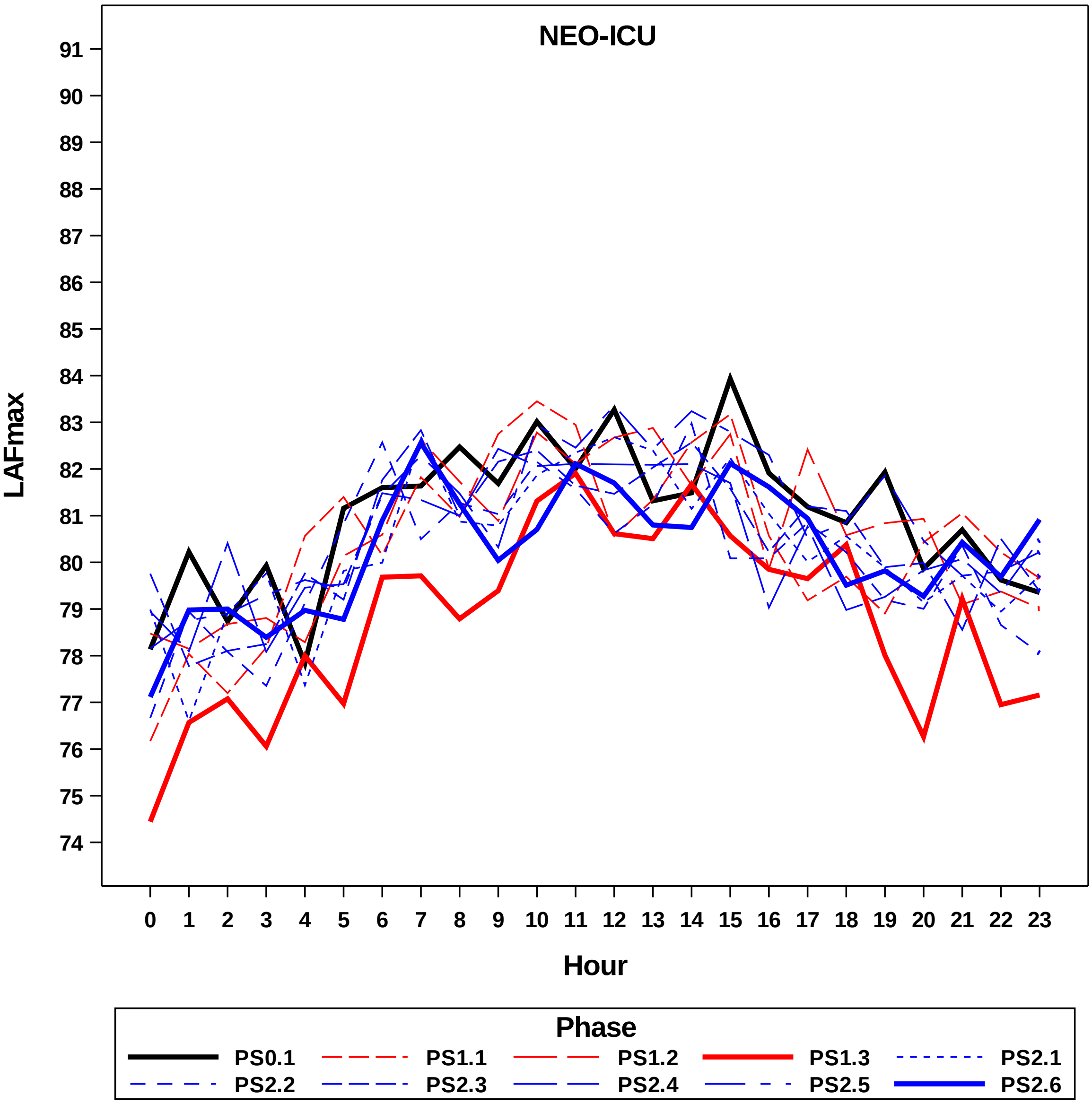

* Neonatal intensive care unit: PS01: baseline; PS13: post-intervention; PS2.6: follow-up; PS1.1, PS1.2, PS2.1, PS2.2, PS2.3, PS2.4; PS2.5: between measuring points

*Figure 6 - Average maximum values LAFmax at ward level (NLO-ICU)**

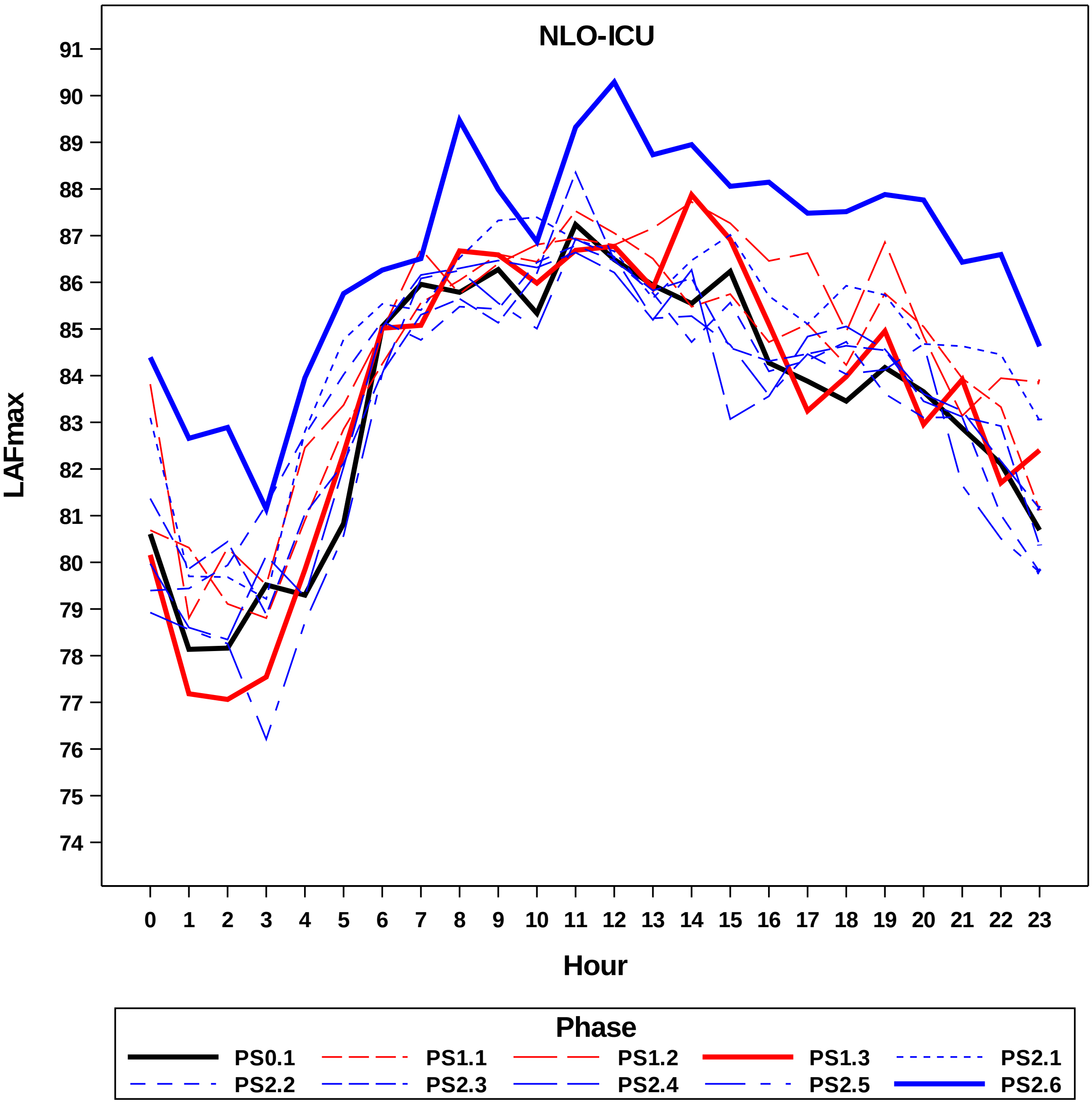

*Neurological intensive care unit: PS01: baseline; PS13: post-intervention; PS2.6: follow-up; PS1.1, PS1.2, PS2.1, PS2.2, PS2.3, PS2.4; PS2.5: between measuring points

Figure 7 - Mean, maximum peak values LCpeakmax baseline and post-intervention

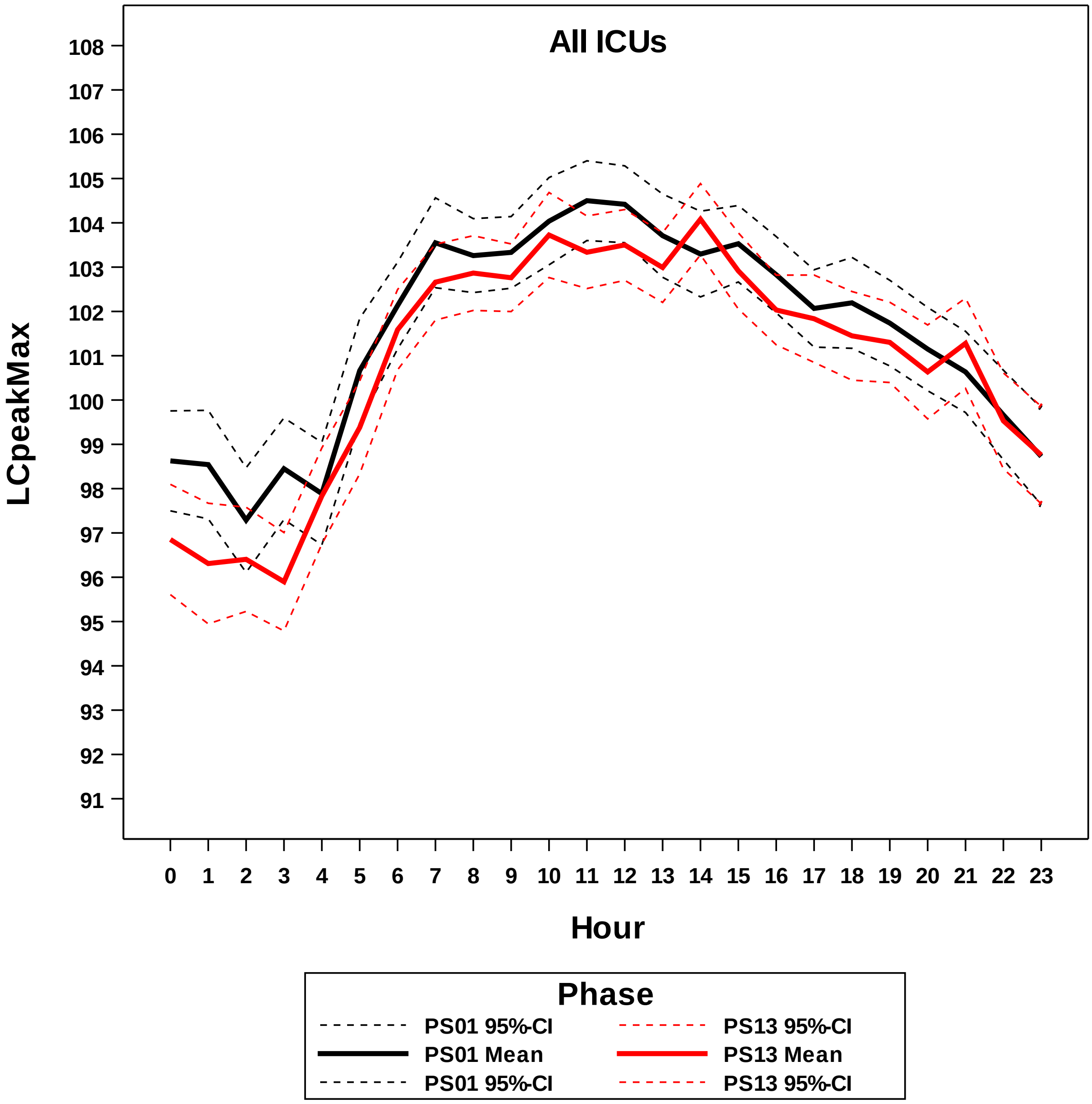

* ICU: Intensive Care Unit, CI: Confidence interval, PS01: baseline; PS13: post-intervention

Figure 8 - Mean, maximum peak values LCpeakmax baseline and post-follow-up

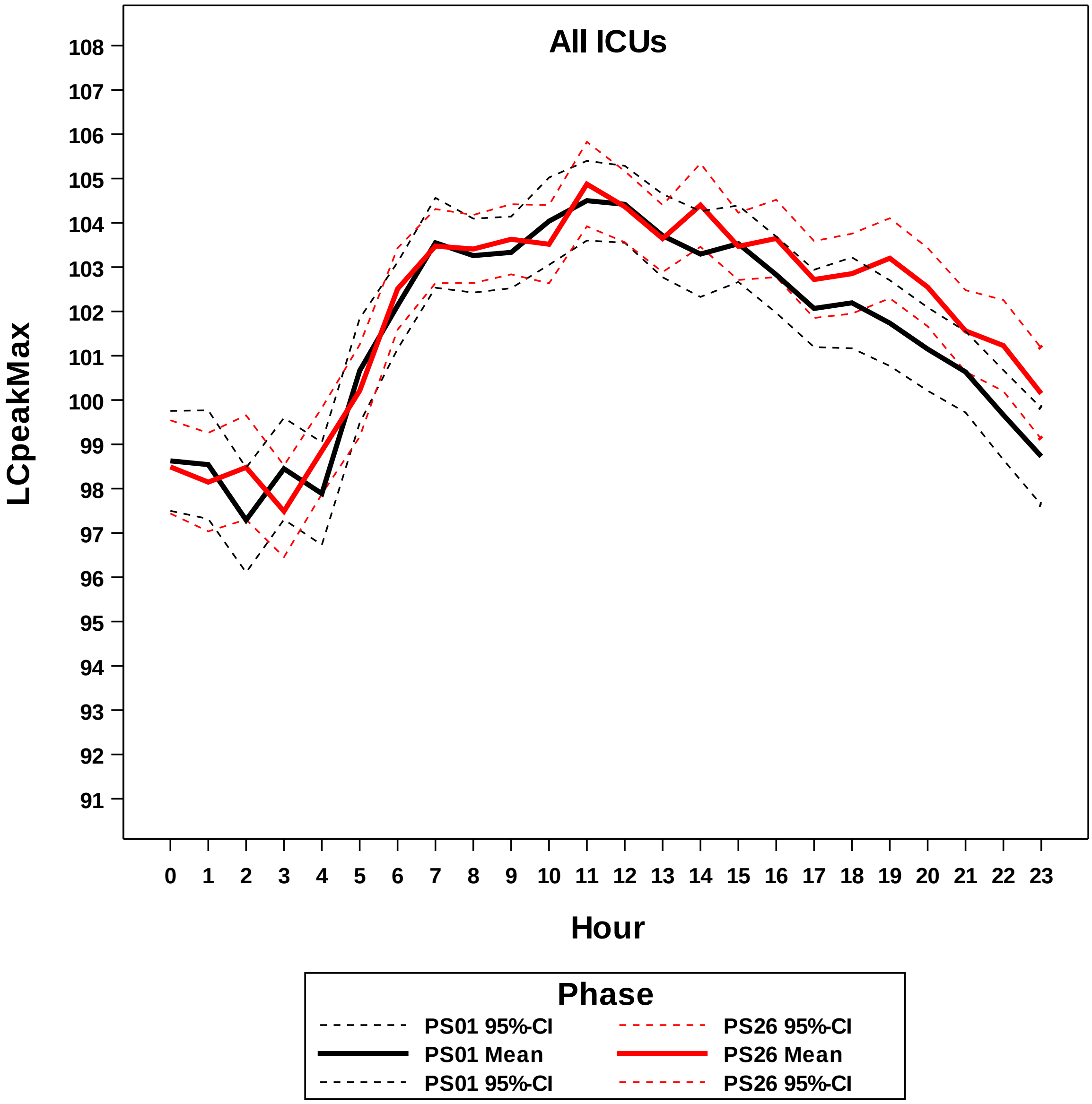

* ICU: Intensive Care Unit, CI: Confidence interval, PS01: baseline; PS2.6: follow-up

Figure 9 - Average, maximum peak values LCpeakmax with all measurement points

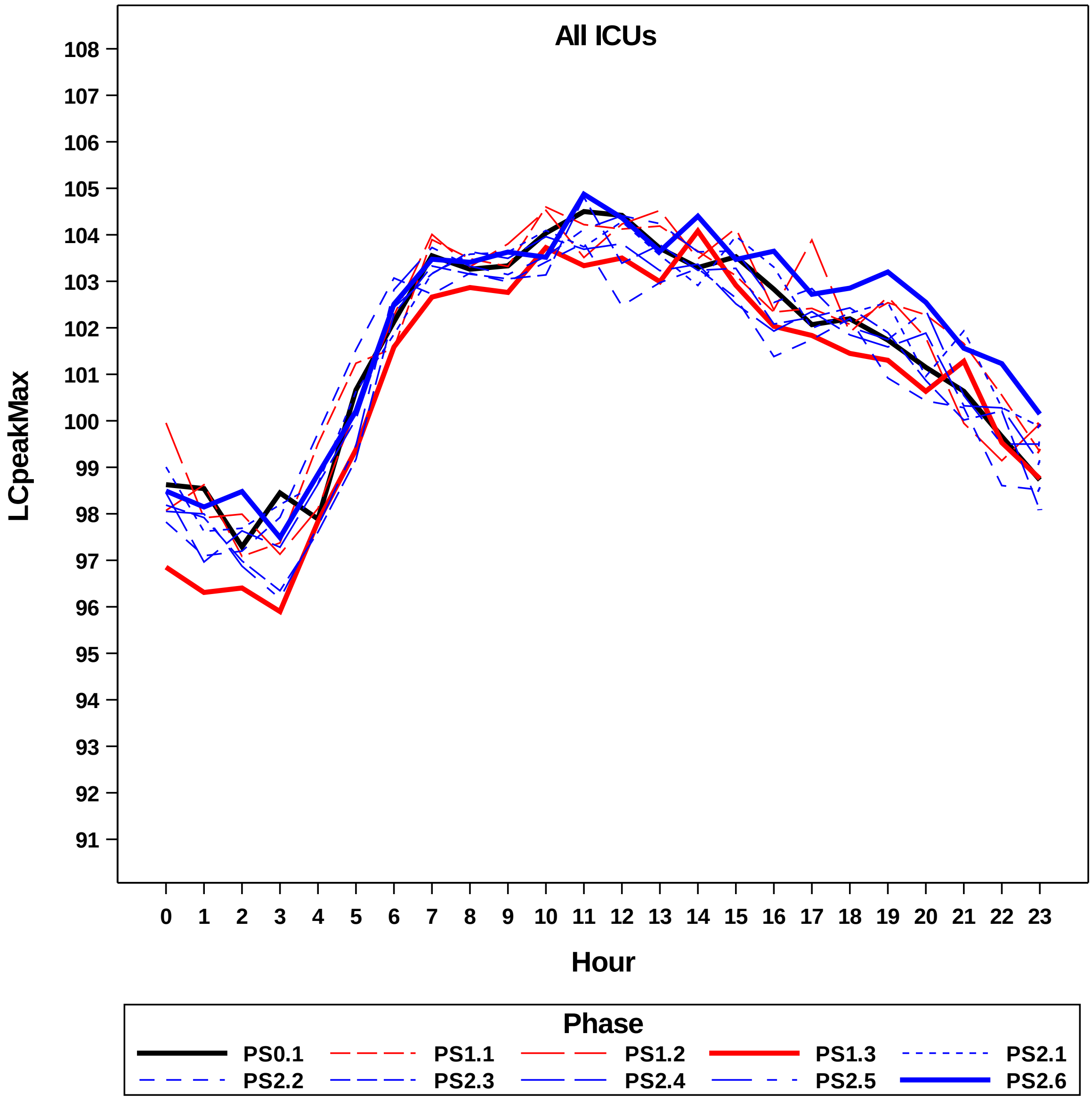

* ICU: Intensive Care Unit; PS01: baseline; PS13: post-intervention; PS2.6: follow-up; PS1.1, PS1.2, PS2.1, PS2.2, PS2.3, PS2.4; PS2.5: between measuring points

*Figure 10 - Average, maximum peak values LCpeakmax of all measurement points at ward level (A-ICU)**

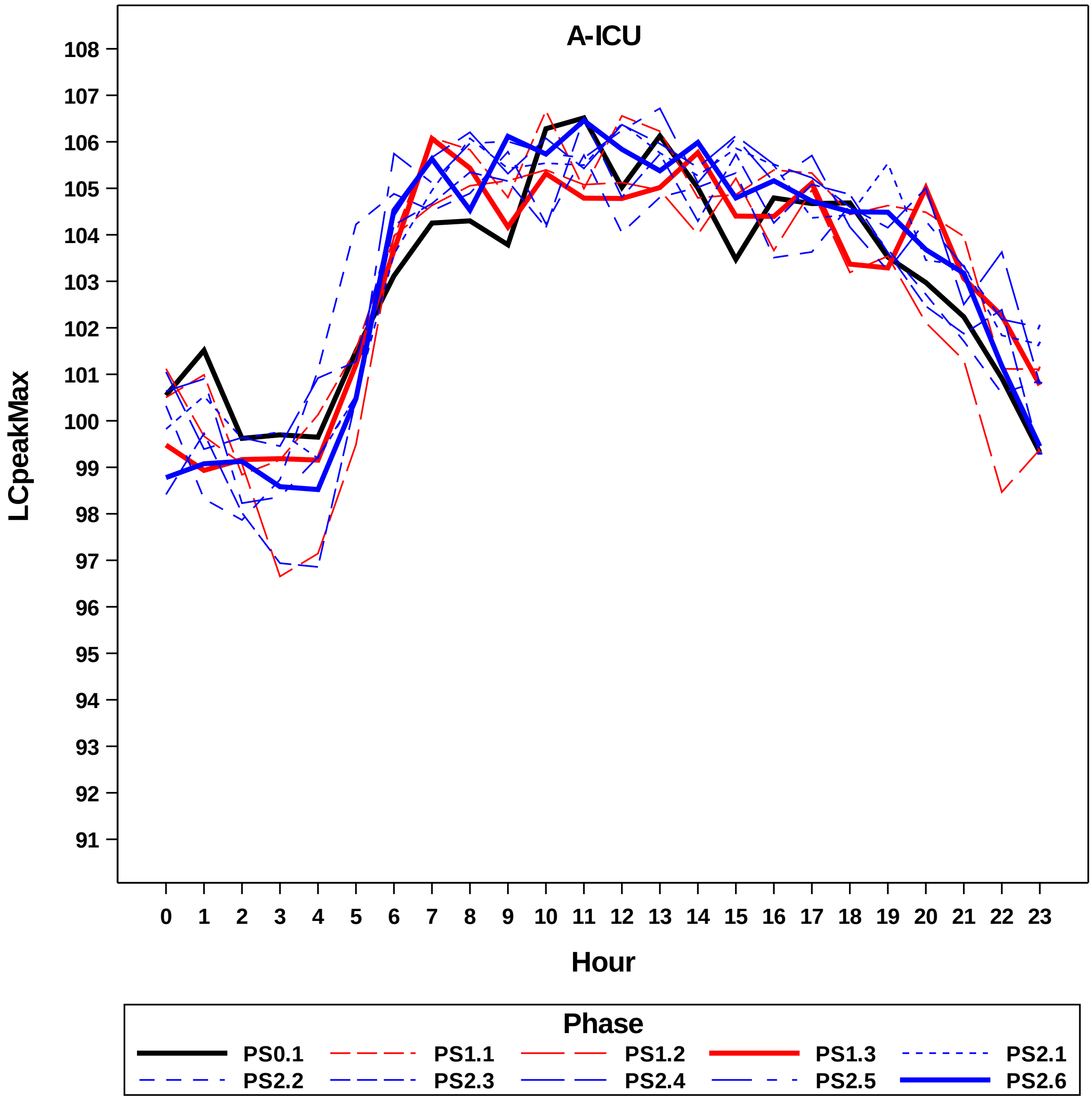

* Anaesthesiology intensive care unit: PS01: baseline; PS13: post-intervention; PS2.6: follow-up;

PS1.1, PS1.2, PS2.1, PS2.2, PS2.3, PS2.4; PS2.5: between measuring points

*Figure 11 - Average, maximum peak values LCpeakmax of all measurement points at ward level (NEO-ICU)**

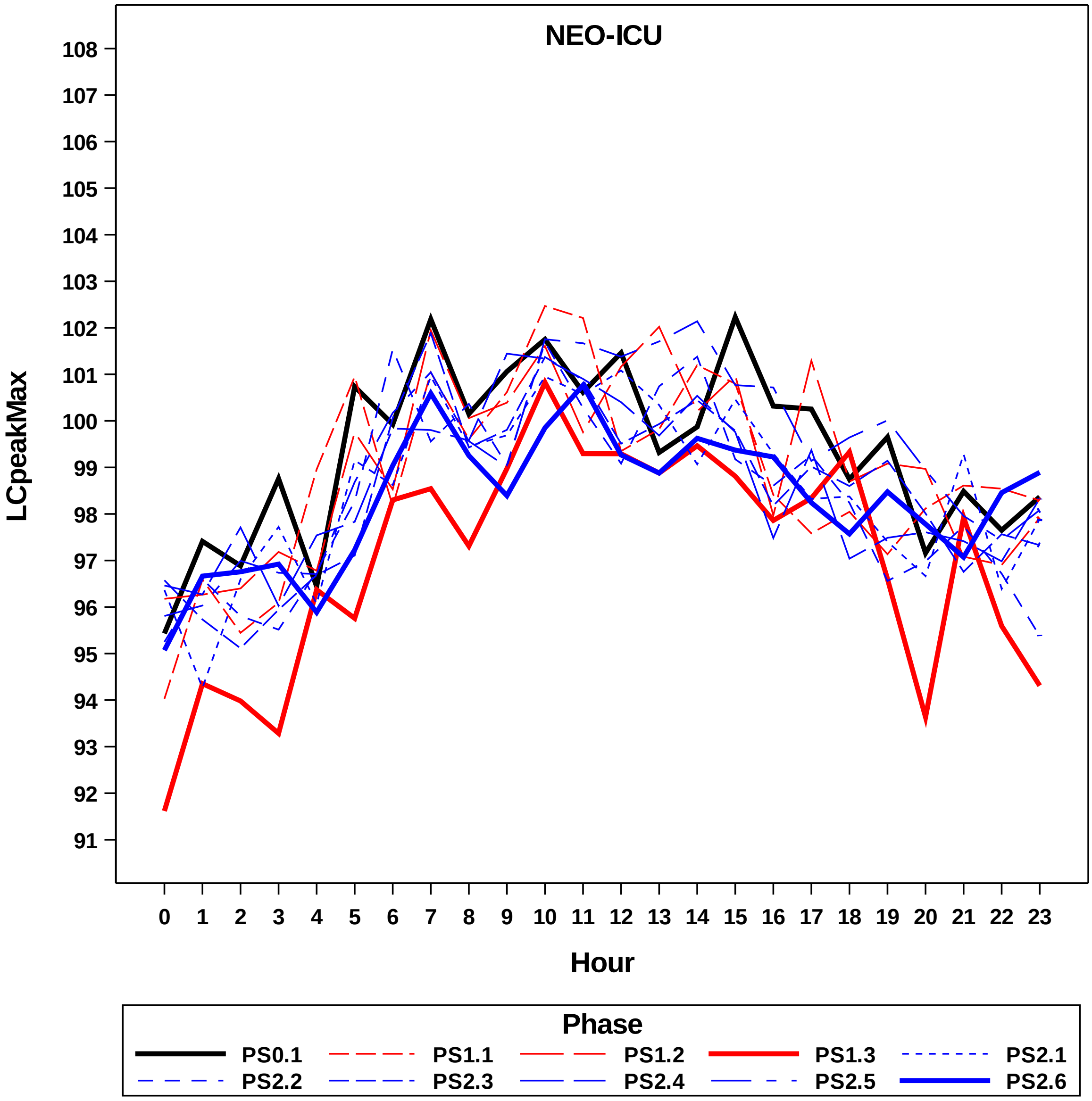

* Neonatal intensive care unit: PS01: baseline; PS13: post-intervention; PS2.6: follow-up; PS1.1, PS1.2, PS2.1, PS2.2, PS2.3, PS2.4; PS2.5: between measuring points

*Figure 12 - Average, maximum peak values LCpeakmax of all measurement points at ward level (NLO-ICU)**

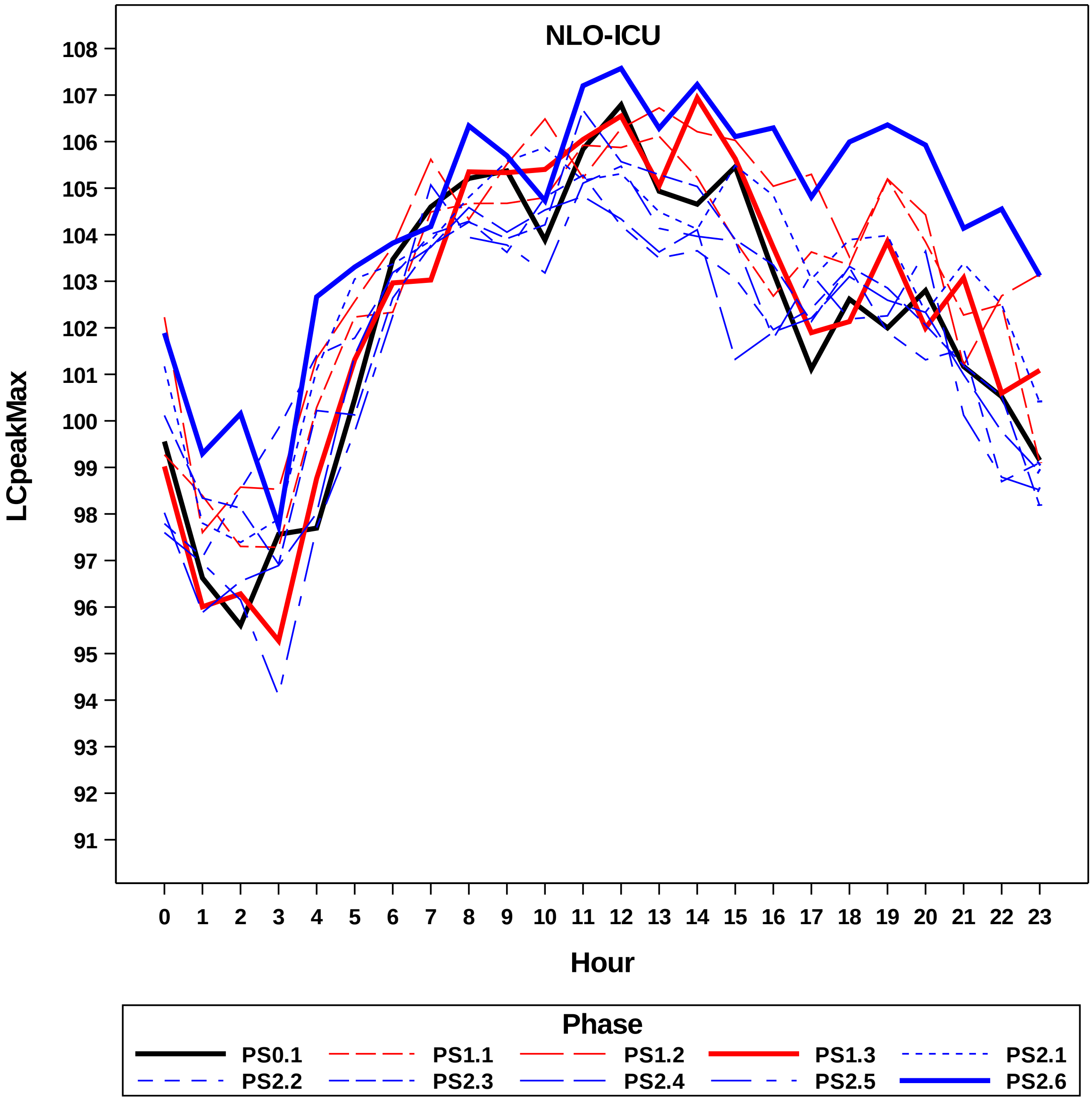

* Neurological intensive care unit: PS01: baseline; PS13: post-intervention; PS2.6: follow-up; PS1.1, PS1.2, PS2.1, PS2.2, PS2.3, PS2.4; PS2.5: between measuring points
